# Polymorphism, Structure, and Nucleation of Cholesterol·H_2_O at Aqueous Interfaces and in Pathological Media: Revisited from a Computational Perspective

**DOI:** 10.1021/jacs.1c10563

**Published:** 2022-03-16

**Authors:** Margarita Shepelenko, Anna Hirsch, Neta Varsano, Fabio Beghi, Lia Addadi, Leeor Kronik, Leslie Leiserowitz

**Affiliations:** † Department of Molecular Chemistry and Materials Science, Weizmann Institute of Science, Rehovoth 7610001, Israel; ‡ Department of Chemical and Structural Biology, Weizmann Institute of Science, Rehovoth 7610001, Israel; § Department of Chemistry, Università degli Studi di Milano, Milano I-20122, Italy

## Abstract

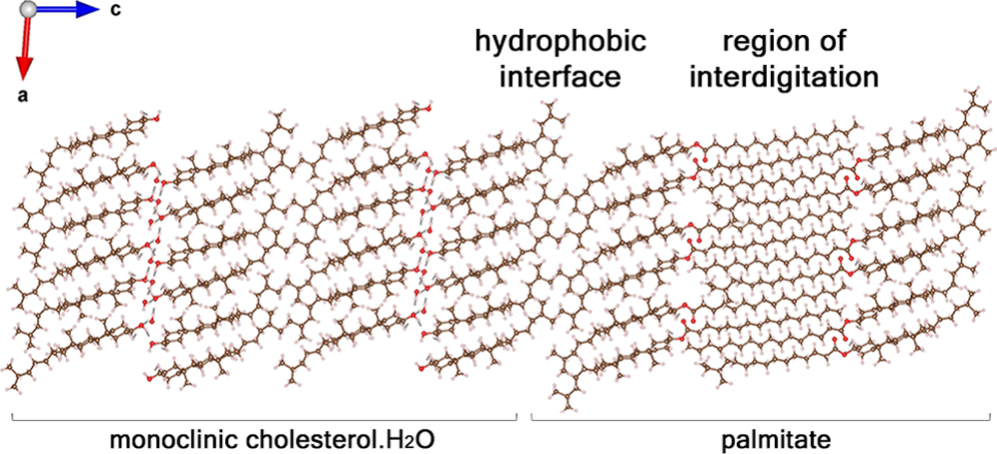

We revisit the important issues of polymorphism, structure, and nucleation of cholesterol·H_2_O using first-principles calculations based on dispersion-augmented density functional theory. For the lesser known monoclinic polymorph, we obtain a fully extended H-bonded network in a structure akin to that of hexagonal ice. We show that the energy of the monoclinic and triclinic polymorphs is similar, strongly suggesting that kinetic and environmental effects play a significant role in determining polymorph nucleation. Furthermore, we find evidence in support of various O–H···O bonding motifs in both polymorphs that may result in hydroxyl disorder. We have been able to explain, via computation, why a single cholesterol bilayer in hydrated membranes always crystallizes in the monoclinic polymorph. We rationalize what we believe is a single-crystal to single-crystal transformation of the monoclinic form on increased interlayer growth beyond that of a single cholesterol bilayer, interleaved by a water bilayer. We show that the ice-like structure is also relevant to the related cholestanol·2H_2_O and stigmasterol·H_2_O crystals. The structure of stigmasterol hydrate both as a trilayer film at the air–water interface and as a macroscopic crystal further assists us in understanding the polymorphic and thermal behavior of cholesterol·H_2_O. Finally, we posit a possible role for one of the sterol esters in the crystallization of cholesterol·H_2_O in pathological environments, based on a composite of a crystalline bilayer of cholesteryl palmitate bound epitaxially as a nucleating agent to the monoclinic cholesterol·H_2_O form.

## Introduction

**Scheme 1:**
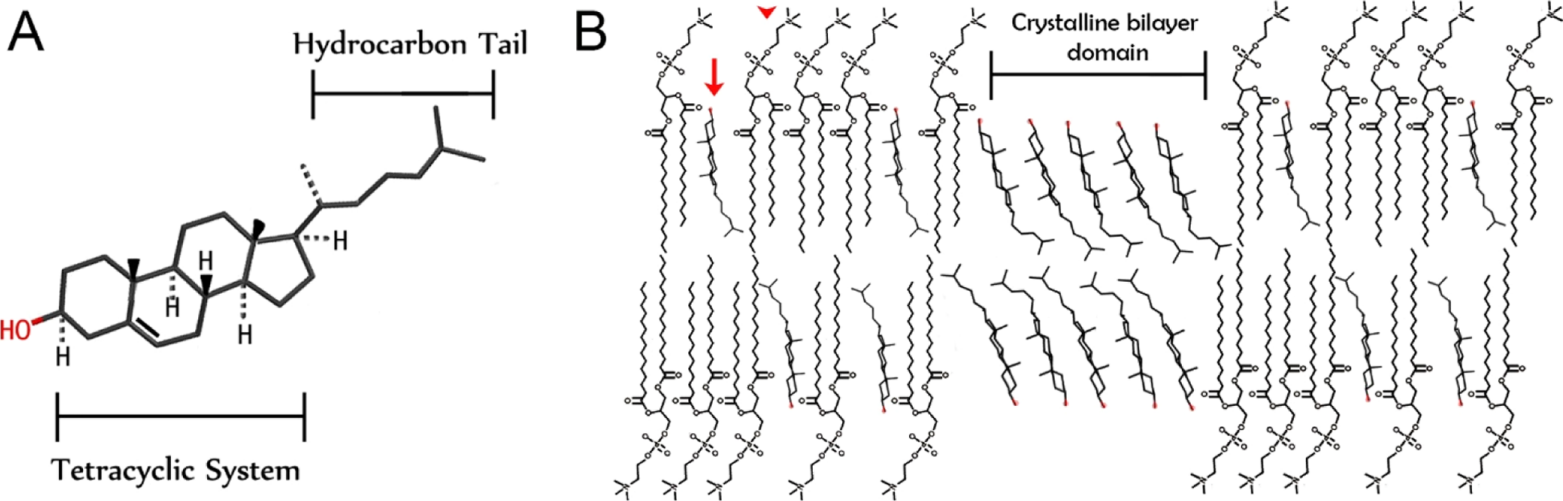
(A) Molecular Structure of Cholesterol; (B) Schematic Representation of a Bilayer Composed of Phosphoglycerolipids (Red Arrowhead) and Cholesterol Molecules (Red Arrow); above a Critical Concentration for Cholesterol, Two-Dimensional (2D) Cholesterol Crystalline Domains are Formed

Cholesterol, the most abundant sterol in mammalian cells, is a vital component of cell membranes and is essential for cell viability.^1^ The cholesterol molecule consists of one hydroxyl group attached to a rigid steroid tetracyclic moiety, terminating with a flexible hydrocarbon chain (Scheme 1A).^2^ Cholesterol is practically insoluble in water. In biological systems, it is mostly solubilized by incorporation in lipid membranes, bile salts, or with lipoproteins in the blood. In cells, most of the cholesterol is located in the plasma membrane,^3^ where the hydrophobic region is embedded alongside the fatty-acid chains of lipids (Scheme 1B), and the hydroxyl group points towards the water molecules surrounding the membrane.

High levels of cholesterol are, however, pathological. They may result in the formation of two-dimensional (2D) crystalline cholesterol domains in cell membranes (Scheme 1B) and ultimately in the precipitation of cholesterol monohydrate crystals.^4,5^ The precipitated cholesterol crystals can hardly be dissolved and therefore accumulate, leading to an increased inflammatory response and severe damage to the tissue.^5−7^ An unfortunate yet common outcome of this cholesterol deposition is atherosclerosis,^8−10^ a major cause of cardiovascular diseases and stroke.

The crystal structure of cholesterol·H_2_O was determined by Craven only as late as 1976,^4^ possibly due to its complexity: the space group is triclinic *P*1, with eight independent cholesterol·H_2_O units per cell, each containing 29 non-hydrogen atoms. We note, nonetheless, that the crystal structure has a high pseudo-symmetry, which was taken advantage of by Craven for structure determination.^11^ The habit of these cholesterol·H_2_O crystals is usually rhomboid plates, by virtue of the crystal pseudo-symmetry and the layer-like molecular packing of cholesterol.

A different cholesterol·H_2_O polymorph with a monoclinic structure was identified in cholesterol nucleation from monolayers and multilayers at the air–water interface.^12,13^ In this process, although the final multilayer structure is generally triclinic, the two leaflets of the first formed cholesterol bilayer are always related by a twofold screw symmetry in a 10 × 7.5 Å^2^ rectangular unit cell. After its initial determination, this polymorph was also detected in supported lipid bilayers composed of lipid mixtures of cholesterol with phosphoglycerolipids and sphingolipids.^14−17^ Two-dimensional monoclinic crystalline domains, formed by segregation of cholesterol from the phospholipids, can either grow into three-dimensional (3D) crystals of the monoclinic polymorph^18^ or transform into the triclinic polymorph.^13,17^ Surprisingly, the monoclinic polymorph was also identified in native bile solutions^19^ related to the formation of cholesterol gallstones and in an atherosclerosis-related cell culture model,^20^ highlighting its possible relevance to cholesterol crystallization in a biological lipid-rich environment, such as that found in cell membranes and bile.

The three-dimensional structure of the monoclinic polymorph was determined making use of thin cholesterol films ranging from 1 to 3 bilayers, in a study on the nucleation of cholesterol at the air–water interface.^12^ Their structures were characterized via synchrotron grazing incidence X-ray diffraction (GIXD), a method that has been applied to molecular assemblies of crystalline films.^21,22^ The single bilayer of cholesterol is of space-group symmetry *p*2_1_, namely the two leaflets are related by twofold screw symmetry.^11^ The space group of the three bilayer film, with unit cell dimensions *a* = 10.2 Å, *b* = 7.6 Å, *c* = 68.2 Å, and β = 94.8°, was shown to be monoclinic *A*2 in a structure determined to near-atomic resolution.^13^ Details on the space group and structure elucidation at its basic level are presented in the Supporting Information S1. The X-ray structure refinement, based on the intensities of 48 (*hkl*) reflections, yielded a satisfactory fit between the observed and computed X-ray structure factors (Figure S1.2), indicating an overall correct structure,^13^ but clearly further refinement is called for in view of the structural assumptions made. Indeed, the H-bonded bilayer sandwiched between cholesterol bilayers shall be shown, in the present study, to have been ill-determined.

Beyond the ambiguous structural details, it is unclear why under some biological and/or chemical conditions, cholesterol grows as the monoclinic polymorph to form 3D structures,^18−20,23^ whereas under other conditions transformation into triclinic plates occurs at an early stage.^13,17^ Understanding this process should be relevant for clarifying critical stages in the pathological crystallization process.

We address these challenges by performing a comprehensive first-principles computational study of both crystal polymorphs of cholesterol·H_2_O, in particular that of the monoclinic form. We obtain a new, fully extended H-bonded network comprising sterol hydroxyl groups and water molecules in a structure akin to that of hexagonal ice. We show that the energy of the monoclinic and triclinic polymorphs is similar, strongly suggesting that kinetic and environmental effects play a significant role in determining polymorph nucleation. Furthermore, we find evidence in support of various O–H···O bonding motifs in both polymorphs that may result in structural disorder. We then rationalize what we believe is a single-crystal to single-crystal transformation of the monoclinic polymorph, on increased interlayer growth beyond that of a single cholesterol bilayer interleaved by a water bilayer. We are also able to explain why, as a single hydrated bilayer, cholesterol crystallizes in the monoclinic form rather than in its triclinic counterpart. We show that the ice-like structure is also relevant to the related cholestanol dihydrate (2H_2_O) and stigmasterol monohydrate (H_2_O) crystals. Finally, we posit a possible role for cholesterol esters in the crystallization of cholesterol·H_2_O in pathological environments, with a composite of a bilayer of cholesteryl palmitate bound epitaxially as a nucleating agent to the monoclinic form of cholesterol·H_2_O.

## Results and Discussion

### Density Functional Theory (DFT) Optimization of the Triclinic Polymorph of Cholesterol·H_2_O

**Figure 1:**
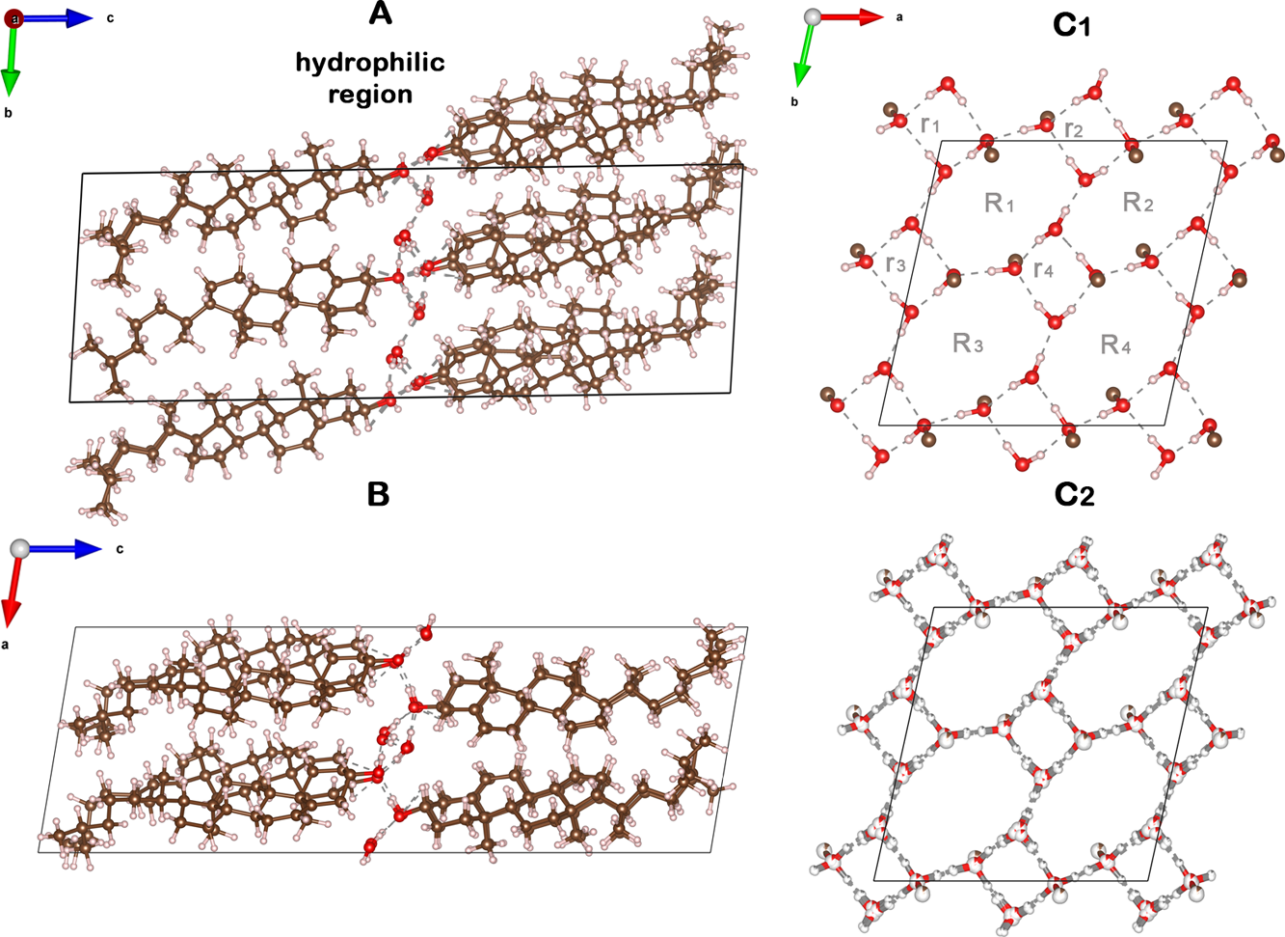
Packing arrangement of triclinic cholesterol·H_2_O, viewed along the: *a*-axis (A), *b*-axis (B), and *c*-axis (C_1,2_). In (C_1,2_), the packing arrangement is limited to the hydrophilic region indicated in A. The atoms are color-coded in white, H; brown, C; and red, O. The different H-bonded rings in panel C_1_ are labeled in grey by *r*_*i*_ and *R*_*i*_, which refer to tetragons and octagons, respectively; the subscript *i* = 1···4 designates the unique polygons of each type. The arrangement in all panels, except for C_2_, is for the lowest energy pseudopolymorph of the triclinic cholesterol·H_2_O. The C_2_ panel arrangement is of the hydrophilic region of the disordered mixture of eight H-bonding networks with partial occupation indicated by partial coloring of pertinent atoms in white. Exclusively in C_2_, H atoms are colored in grey to avoid confusion with the color code used for partial occupation. OH···O bonds are represented as grey dashed lines. The unit cell is delineated by a black rectangle.

We begin our investigation with a dispersion-augmented DFT optimization of the known triclinic structure of the cholesterol·H_2_O crystal (Figure 1). As a starting point for geometry optimization, we use the structure originally determined by Craven,^4,11^ based on the computational refinement of this structure by Frincu et al.,^24^ in which sterol and water H atoms were introduced. There are, however, seven additional ways, beyond the motif originally reported by Frincu et al.,^24^ in which the O–H···O bonds may be arranged in the H-bonding layer. In all arrangements, the basic H-bonding motif is composed of four octagons and four tetragons (Figure 1C_1_), but they differ in the relative orientation of the water molecules and the cholesterol OH group (see Figure S2 for a detailed view of all arrangements). Therefore, crystal structure optimization was performed for all eight structures, with the optimized structural parameters reported in Table S2.1 and average O···O H-bond distances listed in Table S2.2. Computed total energies for the proposed structural motifs, relative to the lowest energy motif (which is found to be different from that of Frincu et al.^24^), are given in Table S2.3. Importantly, all eight H-bonding networks result in very similar energies, suggesting that the crystal structure may be composed of a disordered mixture of them. Furthermore, this structural disorder view agrees well with Craven’s report,^4^ in which the H atoms participating in the H-bonded network were not found in electron density maps and were therefore not included in the X-ray structure factor calculations. Therefore, Figure 1C_2_ presents the disordered-mixture arrangement of the hydrophilic region for these eight H-bonding networks, with the partial occupation indicated by partial coloring of pertinent atoms.

### DFT Optimization of the Monoclinic Crystal Structure of Cholesterol·H_2_O

**Figure 2:**
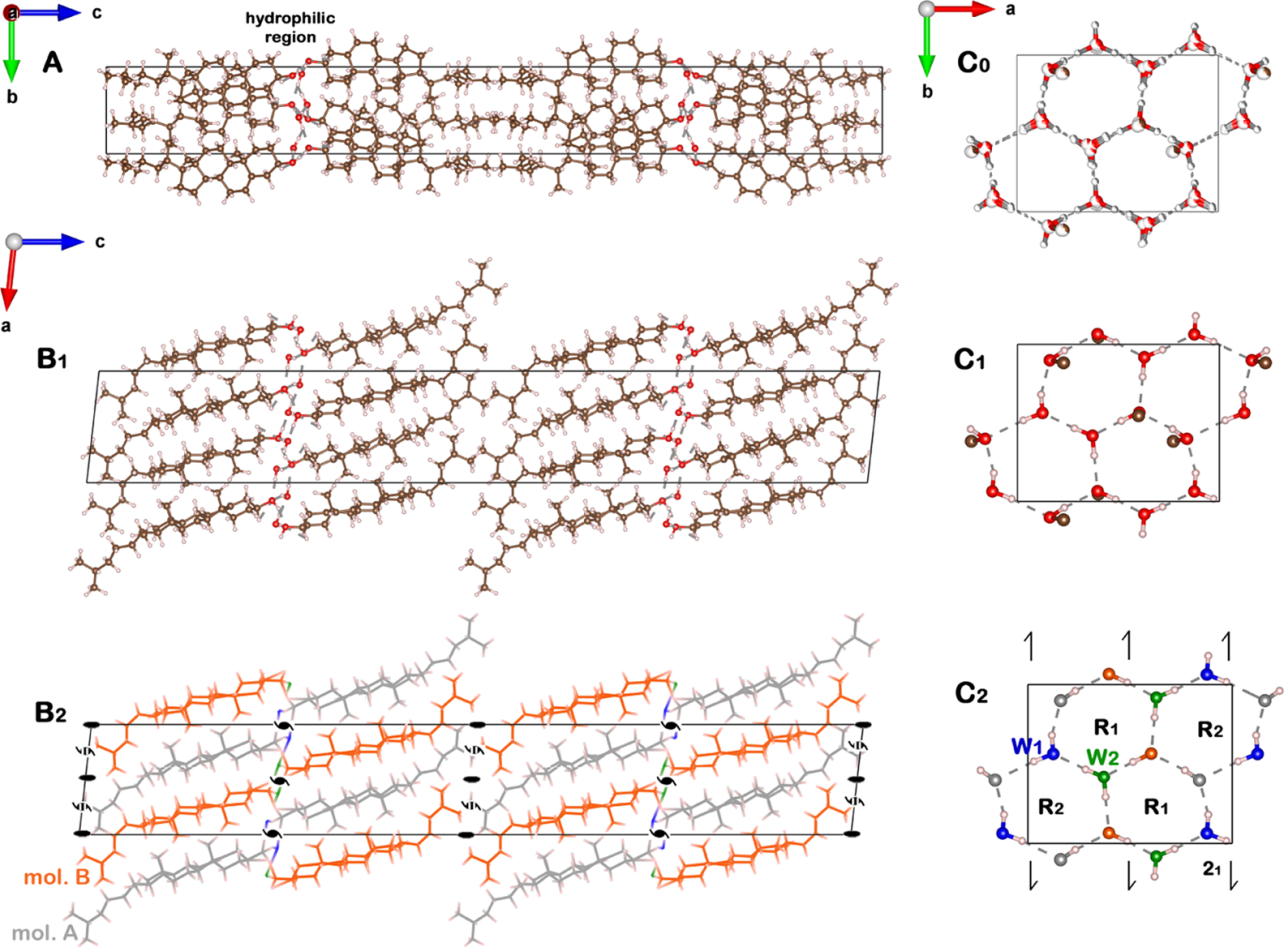
Packing arrangement of the monoclinic cholesterol·H_2_O unit cell, viewed along the: *a*-axis (A), *b*-axis (B_1,2_), and *c*-axis (C_0,1,2_) for the hydrophilic region indicated in (A). For panels (A,B_1_,C_0_,C_1_) the H, C, and O atoms are color-coded in white, brown, and red, respectively. Panels (B_2_,C_2_) present similar views as (B_1_,C_1_), respectively, but with different colors representing different symmetry-unrelated cholesterol and water molecules: grey, cholesterol molecule *A* (mol. *A*); orange, cholesterol molecule *B* (mol. *B*); and blue (W_1_) and green (W_2_), water molecules. The twofold and twofold screw symmetry axes are shown in black. Given that the exocyclic moieties of the non-symmetry related molecules *A* and *B* are part of a pseudo *C*-centered arrangement (see main text), the row of twofold axes along *a* are interleaved by pseudo twofold screw axes (indicated by black and white stripes). The hexagonal H-bonded rings in panel C_2_ are labeled by R_1_ and R_2_. The arrangement in all the panels except for *C*_0_ is for the lowest energy pseudopolymorph of the monoclinic cholesterol·H_2_O. The *C*_0_ panel arrangement is of the hydrophilic region of the disordered mixture of the three most stable H-bonding networks, with partial occupation indicated by partial coloring of pertinent atoms. Exclusively in C_0_, H atoms are colored in grey to avoid confusion with the color code used for partial occupation. OH···O bonds are represented as grey dashed lines. The unit cell is indicated by a black rectangle.

To extend our investigation to the structure of the monoclinic cholesterol·H_2_O polymorph, which incorporates the 10 × 7.5 Å^2^ bilayer motif, we based our initial model on the structure reported by Solomonov et al.,^13^ with H atoms introduced to the cholesterol and water molecules. The obtained structure has eight cholesterol molecules in the unit cell, as shown in Figure 2A,B_1_, and contains two nonequivalent molecules per asymmetric unit (labeled *A* and *B* in Figure 2B_2_). The *c* axis, which is ∼70 Å long, contains two cholesterol bilayers related by the twofold screw (2_1_) symmetry, whereas the two cholesterol leaflets in each bilayer are related by the twofold (2) symmetry.

In order to construct a favorable H-bonding network composed of (cholesterol)-OH and H_2_O molecules in a 1:1 molar ratio, we need to modify the orientation in which the H atoms were originally introduced. To that end, we utilized a model that uses symmetry-related positions of the two asymmetric sterol O atoms, which belong to opposite leaflets of the bilayer (see Supporting Information, S3). These O atoms are separated by ∼3 Å and H-bonded to each other to generate the positions of the symmetry-related water O atoms (Figure S3). This model generates a hexagonal bilayer arrangement of O–H···O bonds, as in hexagonal ice, albeit distorted.

For dispersion-augmented DFT optimization based on the above motif, the monoclinic *A*2 unit cell was reduced to a primitive unit cell that contains half the number of molecular units. Both atomic coordinates and unit cell parameters were then fully optimized.^a^ Finally, the conventional *A*2 unit cell was reconstructed from the global minimum solution, and the optimal H-bonded arrangement was found (see Methods for additional details). Overall, the computationally optimized structure of the monoclinic form given in Figure 2 is very similar to the experimentally determined one. Specifically, the overall atomic structure of the tetracyclic part of the molecules, as well as the molecular tilt relative to the (001) plane, remained essentially unaltered. Some conformational changes occur at the hydrocarbon tail.

The optimized H-bonding motif is composed of two differently shaped fused hexagons, with average O···O H-bond distances of 2.74 Å (Figure 2C_2_, labeled R_1_) and 2.87 Å (Figure 2C_2_, labeled R_2_). There are, however, three other ways in which the O–H···O bonds may be arranged by interchanging the donor and acceptor roles of the sterol oxygens and hydrogen bonding orientation of the acceptor hydrogens (see Figure S4). These three crystal structures were therefore also generated and optimized by the DFT, and their energies are listed in Table S4.1, compared to that of the first-generated motif. The optimized structural parameters thus determined are reported in Table S4.2. Two of these three H-bonding networks are almost as stable as the original motif, suggesting that the crystal structure may be composed of a disordered mixture of three H-bonding networks. Importantly, O···O H-bond lengths of these two motifs are also close to the original motif, while in the fourth motif they differ from the other three (Table S4.2). The hydrophilic region arrangement of the disordered mixture of the three most-stable H-bonding networks, with partial occupation indicated by partial coloring of pertinent atoms, is shown in Figure 2C_0_.

### Comparison of the Two Computed Cholesterol·H_2_O Polymorphs with Experiment

The total energy difference between the most stable pseudopolymorphs of the monoclinic and triclinic cholesterol·H_2_O, as computed using the pair-wise dispersion-augmented DFT (see Methods section), is found to be a small ∼2.25 kcal/mol per molecule, in favor of the triclinic polymorph. While the computed energy ordering is consistent with experiment, it is important to keep in mind that it is at the limit of accuracy of the computational approach.^25,26^ Furthermore, it may be washed out by entropic effects. Therefore, it is indeed reasonable that both polymorphs are accessible experimentally. Further gas-phase calculations of cholesterol molecules taken from the bulk (see Figure S5) reveal that the difference in energy between isolated (gas-phase) cholesterol molecules taken from the bulk of either polymorph without further relaxation is an insignificant ∼0.3 kcal/mol in favor of the triclinic form.^b^ Therefore, the computed energy difference arises entirely from intermolecular interactions.

The optimized structural parameters of the most stable pseudopolymorphs of the monoclinic and triclinic cholesterol·H_2_O are given in Table 1. The results reveal that the computed lattice parameters are consistently smaller than the experimentally determined ones, such that the computed unit cell volume is smaller than the experimental one by ∼9%. This difference between experiment and theory is larger than that typically found with the pair-wise dispersion-augmented DFT for smaller, more rigid molecules (usually <3%,^25,26^ although larger volume discrepancies of ∼5% have been reported for flexible molecules).^26^ To test whether this is a consequence of the level of theory used, we performed further optimization using the more advanced many-body dispersion (MBD) approach, with^27^ and without^28^ non-local corrections (see Methods section), for the monoclinic polymorph. This, however, did not result in any significant improvement of agreement with the experiment (see Table S6).

**Table 1:** Experimental and Calculated Unit Cell Parameters (in Å, Degrees, and Å^3^), Derived via DFT for the Triclinic and Monoclinic Forms of Cholesterol·H_2_O^a^

method	*a*	*b*	*c*	α	β	γ	*V*
SC-XRD^4^	12.39	12.41	34.36	91.9	98.1	100.8	5128.2
PBE-TS	11.85	11.95	34.16	92.1	98.8	102.0	4663.3
Δ in %	4.34	3.73	0.60	0.2	0.7	1.2	9.1
GIXD^13^	10.15	7.57	68.20	90.0	94.8	90.0	5222.0
PBE-TS	9.63	7.46	66.90	90.0	96.3	90.0	4778.7
Δ in %	5.09	1.45	1.90	0.0	1.6	0.0	8.5

a Experimental parameters for these two forms were taken from Craven^4^ and Solomonov et al.^13^ We compare them to the corresponding lowest energy H-bonding motifs optimized by the DFT.

**Figure 3:**
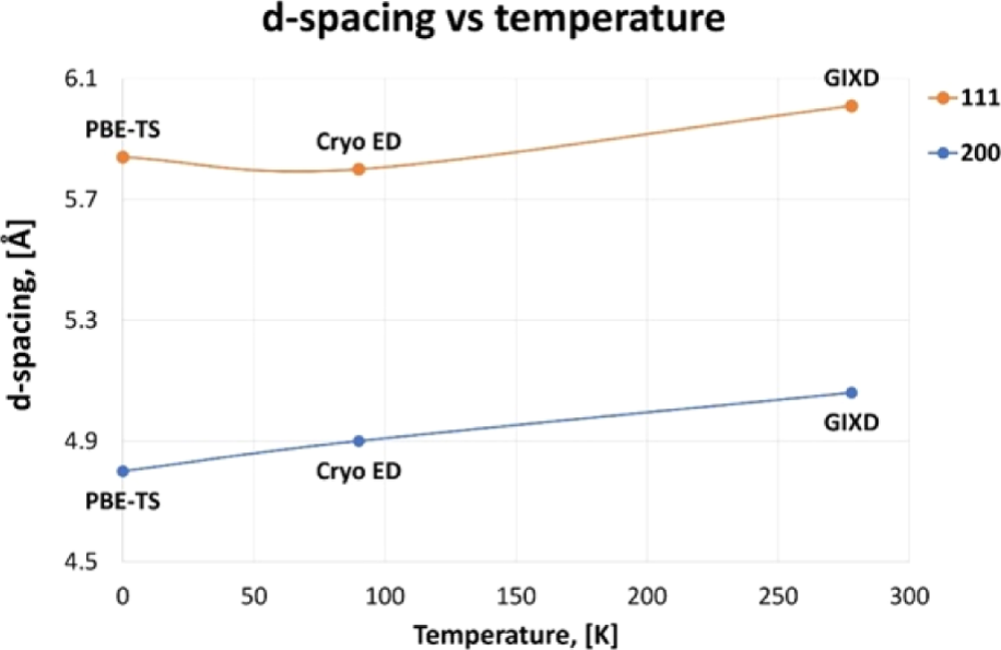
Temperature dependence of the *d*-spacing of monoclinic cholesterol·H_2_O measured by electron diffraction (ED)^19^ and grazing incidence X-ray diffraction (GIXD)^13^ and compared to calculations at the PBE-TS level of theory.

An alternative explanation for this discrepancy is that the exocyclic moiety of cholesterol in the monolayer at the air–water interface,^12^ as well as in the triclinic polymorph of cholesterol·H_2_O, is characterized by large thermal motion at room temperature.^4,11^ Specifically, the proposed trigonal lattice symmetry and packing arrangement of the cholesterol crystalline monolayer on water^12^ have been rationalized in terms of pronounced libration about the long molecular axis. This molecular motion is high enough to exclude the contribution of the exocyclic hydrocarbon moiety to the Bragg rod intensity profile as measured by GIXD.^12^ As for the thermal motion of the eight cholesterol molecules in the triclinic monohydrate,^11^ the ratio between the average atomic displacement parameter (ADP) of the flexible exocyclic group and of the rigid cyclic system is 2.5, with a maximum ratio of 4. Thus, libration of the molecule around its long axis, coupled with motion of the exocyclic hydrocarbon moiety, may induce increased intralayer packing distances in the monoclinic and triclinic polymorphs. In this view, the discrepancy between the theory and experiment then arises mostly from the comparison of room temperature experimental data with 0 K computational data. Theoretically, such thermal effects can be tested within DFT using first-principles molecular dynamics.^29^ However, for the large unit cells studied here, this would be prohibitively expensive. Instead, we address the issue from the experimental perspective by comparing the computed data against (*hkl*) (111) and (200) *d*-spacings, which are deduced from the electron diffraction (ED) measurements of Weihs et al.,^19^ corresponding to a temperature of 90 K, and from the GIXD data of Solomonov et al.^13^ taken at 278 K. Overall, this comparison, summarized in Figure 3 and Table S7, shows a reduction of ∼3.0–5.5% in the *d*-spacing with decreasing temperature. While a detailed temperature dependence is not available experimentally, this reduction in *d*-spacing is consistent with the difference between the theory and experiment.

We note that we have also compared the theoretical growth morphologies of the triclinic and monoclinic structures, determined using interatomic potential energy computations. The method and results are given in the Supporting Information (S8) and again reveal, by and large, a match to those of the observed morphologies of crystals grown in solution.

Taken together, the above comparison validates the computational approach, such that we can draw new insights from further calculations.

### Structure of Monoclinic Cholesterol·H_2_O on Early Growth and Its Preference as a Single Bilayer

The two cholesterol leaflets are related by twofold screw symmetry for a single bilayer at the air–water interface or else hydrated at both sides of the film. However, as a triple bilayer crystal on the water surface, the cholesterol leaflets, in contact via their hydrocarbon tails, are related by twofold symmetry.^13^ At first sight, this is surprising since, in general, the twofold screw symmetry element lends itself to better molecular packing than the twofold symmetry.^30^

To rationalize this observation, we generated a series of hypothetical *bulk P*2_1_ crystal structures of cholesterol·H_2_O (Figure S9.1) by first replacing the twofold axes of the *A*2 polymorph with twofold screw axes. This change in the space group was followed by offsetting adjacent cholesterol bilayers along the *a*-axis but maintaining the original H-bonded bilayer system, across which the corresponding cholesterol layers are related by the twofold screw symmetry. The DFT-computed energy profile of this series of generated crystal structures (Figure S9.2) revealed that the crystal structure with no offsetting of the adjacent cholesterol bilayers along the *a*-axis is the most stable. We then compared this hypothetical bulk *P*2_1_ structure of cholesterol·H_2_O, after relaxation, with the above-obtained *A*2 polymorph. The comparison shows an energy difference between the two structures, at the MBD level of theory, of ∼1 kcal/mol per molecule in favor of *P*2_1_. Note that the computations correspond to the structures at 0 K, such that this small difference is of the order of the energy associated with thermal fluctuations at room temperature. This consideration suggests that temperature may be a determining factor in the preferred stability of the *A*2 motif at room temperature. This preference is consistent with the crystal structure of stigmasterol monohydrate, which crystallizes at room temperature in the monoclinic *P*2_1_ motif (*a* = 10.27 Å, *b* = 7.63 Å, *c* = 35.39 Å, and β = 94.4°).^31^ The exocyclic moiety of this sterol (see Figure S13A) contains a >C=C< double bond that makes it less flexible than the exocyclic group of cholesterol, which is in turn responsible for the contact between layers of the two non-symmetry-related cholesterol molecules *A* and *B* (see Figure 2B_2_). The vector distance between the centers of mass of the (terminal) atoms of the exocyclic moieties of molecules *A* and *B* is very close to 0.5(*a* + *b*) (Table S10.2), namely, it is a property of a pseudo *C*-centered arrangement of exocyclic groups. Therefore, the exocyclic groups make interlayer contact via a combination of alternating twofold (2) and pseudo twofold screw (2_1_) axes along the *a*-axis, as shown in Figure 2B_2_. It is also noteworthy that in the cholesteryl ester crystal structures, which incorporate the monoclinic 10 × 7.5 Å^2^ motif, both types of cholesterol bilayer arrangements, namely interlayer contact via twofold or twofold screw symmetry, have been observed (see Table 6.1 in ref (2)).

The next question to examine concerns the energetics of a *single* cholesterol bilayer, because the symmetry of this bilayer at the air–water interface or hydrated at opposite sides is *p*2_1_, as is also the trilayer of the stigmasterol hydrate.^12^ We therefore generated a bilayer in which the two leaflets are related by twofold screw symmetry, as opposed to a twofold axis (Figure S11A,B). The computed energy difference between both relaxed structures in vacuum of the *p*2_1_ bilayer, compared to the bilayer with the original *p*2 symmetry, at the MBD level of theory, was found to be ∼0.85 kcal/mol per molecule, with *p*2_1_ being consistently more stable.

The computed stability of the *p*2_1_ cholesterol bilayer prior to growth beyond the single bilayer agrees with the experiment and our deduction of a single-crystal to single-crystal transformation on further growth beyond the first cholesterol bilayer. This change essentially involves an interlayer shift of *b*/2 such that the two cholesterol leaflets become related by twofold instead of twofold screw symmetry. This transition is consistent with the similarity of the Bragg rod data of the one, two, and three cholesterol bilayer films at the air–water interface (see Figure S1.1).

Finally, we tackled the question of why, as a hydrated single cholesterol bilayer, the monoclinic *p*2_1_ form is always observed, as opposed to a single bilayer of the triclinic *p*1 counterpart (Figure S11C). The energy difference between the *p*2_1_ and *p*1 structures in vacuum upon relaxation at the MBD level of theory is insignificant, at ∼0.15 kcal/mol per molecule in favor of the triclinic *p*1 polymorph. This result suggests that the observed preference for the monoclinic polymorph is not dictated by differences in the properties of the bilayer as such, but rather by hydration.

### Generality of the Ice-Like Motif in Sterol Crystal Structures Embodying the 10 × 7.5 Å^2^ Motif

**Figure 4:**
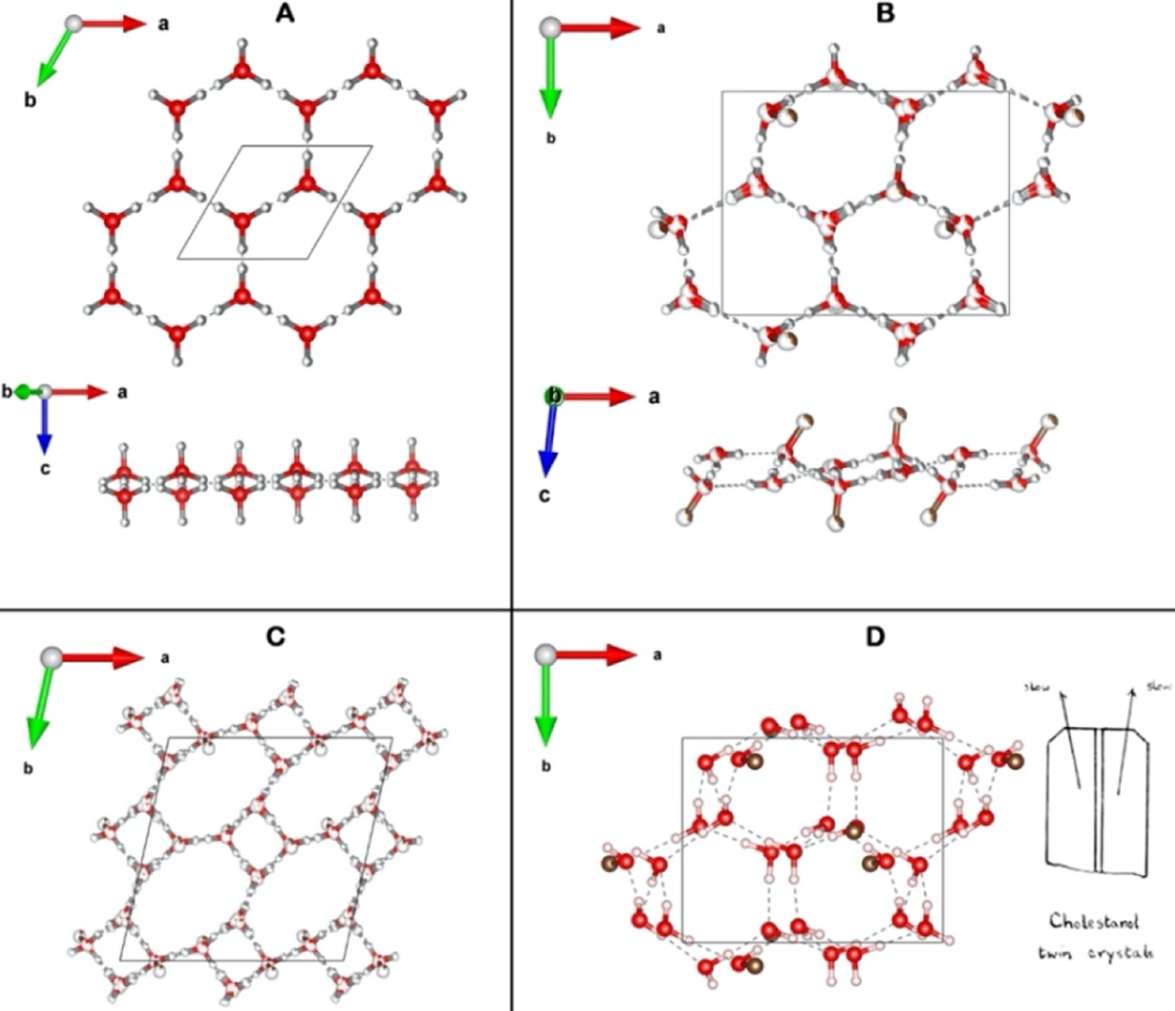
Views of the H-bonded bilayers of cholesterol·H_2_O and cholestanol·2H_2_O. Top and side views of (A) Hexagonal H-bonding bilayer in the structure of hexagonal ice, in which the O–H···O bonds are proton disordered^36^ and (B) disordered mixture of the three most stable H-bonding networks of monoclinic cholesterol·H_2_O. (C) Disordered mixture of the 8 H-bonding networks of triclinic cholesterol·H_2_O. (D) Model H-bonded network of cholestanol·2H_2_O. Also shown is a drawing of cholestanol·2H_2_O crystals taken from the PhD thesis of D. Hodgkin (1937),^34^ reproduced by permission of the Hodgkin family. The two crystals are elongated, twinned about the [010] direction, and show mainly the (001) face with minor (100), (010), and (110) side faces. The C and O atoms are color-coded brown and red, respectively, with partial occupation indicated by partial coloring of pertinent atoms. In panels (A–C), H atoms are colored in grey to avoid confusion with the color code used for partial occupation.

Clearly, the identification of an ice-like motif (Figure 4A) plays a major role in the above considerations for the monoclinic polymorph (Figure 4B). This arrangement is quite distinct from the hydrogen-bonded network in the triclinic system (Figure 4C), although in both each oxygen atom participates in three H-bonds of a proton-disordered network. The ice-like network can be rationalized in view of a partial lattice and stereochemical match to that of hexagonal ice. Specifically, the monoclinic motif incorporates a pseudo-centered 7.5 × 5 Å^2^ sub-lattice. Indeed, ice nucleation was promoted via monolayers of long-chain aliphatic alcohols, which are packed in a two-dimensional 7.5 × 5 Å^2^ lattice and whose OH groups are also arranged in a (pseudo) centered cell,^32,33^ as in the layer structure of hexagonal ice itself. It is therefore important to examine whether the above-suggested ice-like motif is unique to the monoclinic structure of cholesterol·H_2_O or is more general.

The 10 × 7.5 Å^2^ monoclinic motif is predominant in the two- and three-dimensional crystals of various sterols.^13^ Indeed, the 10 × 7.5 Å^2^ axial system is also found in the cholestanol·2H_2_O^34,35^ crystal, a close relative of cholesterol. In particular, the unit cell of cholestanol dihydrate, reported in the Ph.D. thesis of D. Hodgkin^34^ to be of triclinic *P*1 symmetry, can be transformed to a pseudo *A*2 cell similar to that of monoclinic cholesterol·H_2_O but with a *c*-axis longer by approximately 6 Å, which is consistent with a double ice-like bilayer (Table 2).^32,33^ To test this, we generated a model packing by inserting two additional H-bonded bilayers to the monoclinic structure of cholesterol·H_2_O, converting cholesterol to cholestanol, and optimizing the crystal structure by the DFT. A stable structure with *A*2 symmetry and dimensions close to the experiment (Table 2) were indeed found, lending further substantial support to the suggested ice-like structure and demonstrating its generality. The H-bonding motif and drawings of the crystal shape of cholestanol·2H_2_O are given in Figure 4D, with the energy-minimized structure provided in Figure S12.

**Table 2:** Summary of Structural Parameters of Cholestanol·2H_2_O (in Å, Degrees, and Number of Molecules per Asymmetric Unit, *n*) as Compared to Monoclinic Cholesterol·H_2_O

	*a*	*b*	*c*	α	β	γ	space group	*n*
triclinic cell^34,35^	9.79	7.76	36.8	83.0	106.0	88.5	*P*1	4
triclinic, transformed to pseudo *A*2^13^	9.8	7.8	73.8	89.1	106.1	88.5	pseudo *A*2	2
DFT-optimized *A*2	9.52	7.44	75.14	90.0	95.3	90.0	*A*2	2
monoclinic cholesterol·H_2_O^13^	10.15	7.57	68.20	90.0	94.8	90.0	*A*2	2

**Figure 5:**
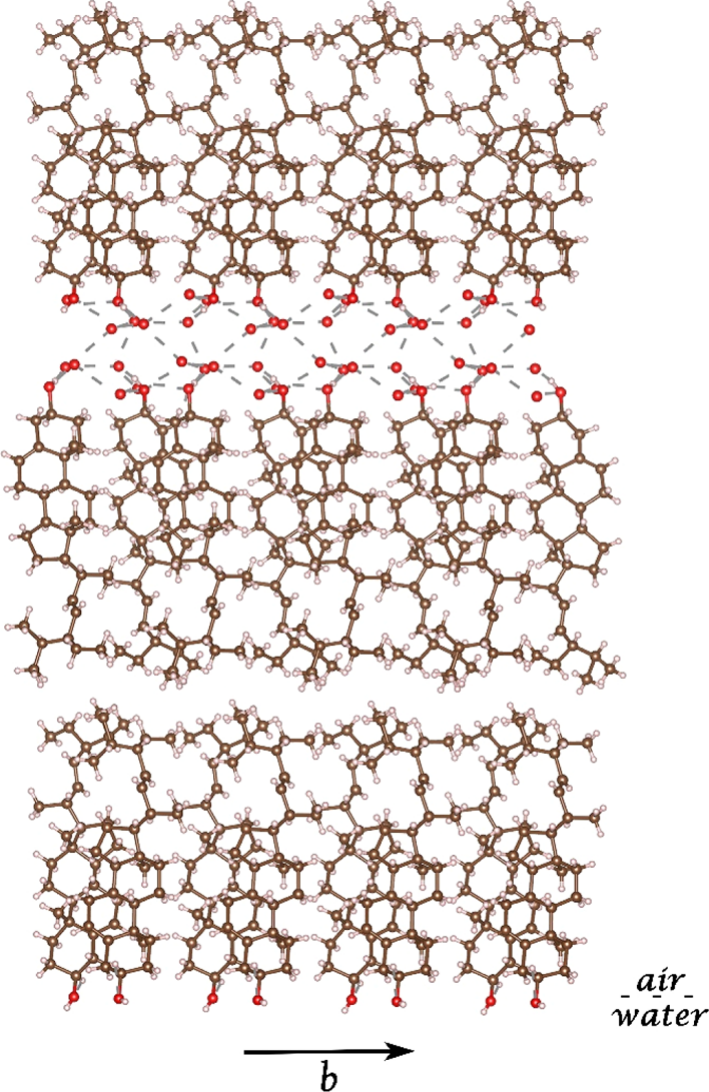
Model of the trilayer packing arrangement of stigmasterol hydrate on a water surface, based on an analysis of GIXD measurements thereof^12,37^ (Figure S14), on the sigmasterol·H_2_O crystal structure^31^ (Figure S13B,C), and on the model structure of cholestanol dihydrate (Figures 4D and S12). The trilayer structure viewed along the *a*-axis incorporates an ordered layer of water molecules whose ice-like hydration structure is probably a double bilayer similar to cholestanol dihydrate. Both structures embody the 10 × 7.5 Å^2^ monoclinic 2_1_ motif.

We note that the ice-like motif also occurs in the three-dimensional crystal structure of stigmasterol·H_2_O^31^ alluded to above (Figure S13C) and, in all probability, in the trilayer film of stigmasterol hydrate (*a* = 10.2 Å and *b* = 7.7 Å, *p*2_1_), as suggested in Figure 5. The results of a GIXD^12,37^ characterization of such a stigmasterol trilayer formed at the air–water interface are shown in Figure S14.

### Model of Induced Nucleation of Monoclinic Cholesterol·H_2_O via a Bilayer of Cholesteryl Palmitate

**Scheme 2:**
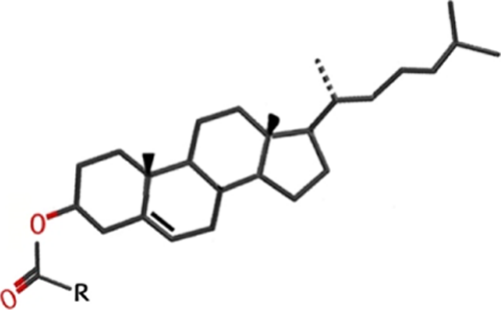
Cholesteryl Esters The palmitate derivative has a saturated aliphatic chain (C_16_H_31_); the chain of the oleate derivative (C_18_H_33_) contains a C=C double bond with a cis configuration; the linoleate has a chain (C_18_H_31_) with two C=C double bonds, both with the cis configuration.

Last but not at all least, we finally address the possible relevance of our results to the nucleation and growth of cholesterol crystals in atherosclerosis. In addition to cholesterol crystals, most atherosclerotic plaques develop calcifications of apatite (calcium phosphate) crystals. Lonsdale published a paper in 1968^38^ in which she laid out possible epitaxial matches and, consequently, epitaxial growth of crystals in gallstones. Could the same apply to atherosclerosis? We consider here the possibility that epitaxy may play a role in the nucleation of cholesterol monohydrate crystals, not in relation to apatite, but to cholesteryl esters. Cholesterol crystal deposition occurs in atherosclerosis when cholesterol reaches super-saturation in the lipid environment because of the accumulation of unesterified cholesterol following hydrolysis of cholesteryl esters. The hydrolysis of cholesteryl esters is associated with the breakdown of lipid bodies inside lysosomal compartments or at extracellular locations following cell death.^8,39,40^ The major cholesteryl esters found in atherosclerotic lesions are cholesteryl palmitate, oleate, and linoleate^5,41^ (Scheme 2). Of these three molecules, the palmitate crystallizes in the rectangular 10 × 7.5 Å^2^ cholesterol-type monoclinic motif (as does the related myristate derivative, see Supporting Information S1); a crystal structure of the linoleate has not been reported, perhaps because of its doubly unsaturated chain; the oleate derivative has a crystal structure with no resemblance to that of monoclinic cholesterol·H_2_O.^42^

**Figure 6:**
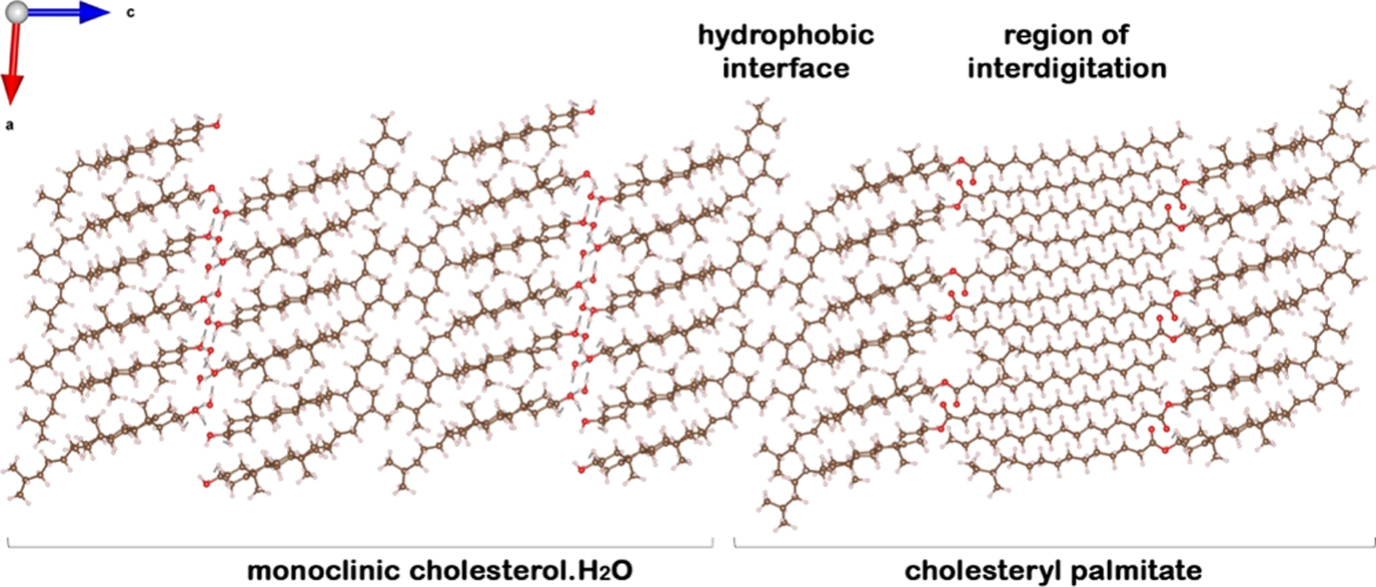
Simulation of a composite crystal in which the cholesteryl palmitate interdigitated bilayer is epitaxially bound to monoclinic cholesterol molecules across twofold axes. As a bilayer, cholesteryl palmitate is found to be sufficiently stable and could therefore nucleate the monoclinic crystals of cholesterol·H_2_O by epitaxy.

According to surface pressure–area isotherms and grazing incidence X-ray diffraction measurements, cholesteryl tridecanoate (C_13_H_25_), palmitate (C_16_H_31_), and stearate (C_18_H_35_), each containing saturated aliphatic chains, self-assemble into crystalline single bilayer films in a unit cell of 10 × 7.5 Å^2^ at the air–water interface, where the two layers are interdigitated across their hydrocarbon ester chains.^43^ The cholesteryl moieties of the bilayer form a structure akin to the layer of cholesterol molecules in its monoclinic polymorph. Given the ease with which cholesteryl palmitate forms a single crystalline bilayer at the air–water interface,^43^ we propose a model by which the palmitate derivative forms a crystalline interdigitated bilayer inside lipid bodies or lysosomal compartments and acts as a nucleating agent of the monoclinic cholesterol form. We therefore set out to examine, by DFT, the possibility of epitaxial nucleation of cholesterol·H_2_O onto the cholesteryl surface of a cholesteryl palmitate bilayer. Making use of the crystal structure of cholesteryl myristate,^44^ we have been able to construct a model of the packing arrangement of a bilayer of cholesteryl palmitate, bound epitaxially as a nucleating agent to the monoclinic form of cholesterol·H_2_O (Figure 6). We have been able to converge this structure to atomic forces smaller than 10^–2^ eV/Å, meaning that it is at least locally stable, thereby lending support to the model hypothesis.

### Transformation from the Monoclinic to the Triclinic Form of Cholesterol·H_2_O

The different stages of cholesterol formation at the air–water interface are depicted in Figure S15. The final cholesterol transformation from monoclinic *A*2 to triclinic *P*1 space group symmetry, involving a lattice change from 10 × 7.5 Å^2^, γ = 90° to 12.4 × 12.4 Å^2^, γ = 101° has been modeled as a single-crystal to single-crystal layer transition, at least in its initial stages.^13^ Experimentally,^12−16^ for a single bilayer of cholesterol hydrated at both sides, the monoclinic *p*2_1_ arrangement is more stable. Computationally, this bilayer is as stable as that of the triclinic *p*1 form, which suggests that hydration tips the balance in favor of the monoclinic bilayer. The unit cell of the triclinic polymorph contains eight non-symmetry-related molecules with essentially four different exocyclic group conformations, whereas the monoclinic structure contains two symmetry-unrelated cholesterol molecules with different conformations of their exocyclic moieties. Therefore, the greater stability of the triclinic polymorph at room temperature may be explained in terms of it having more degrees of freedom than the monoclinic polymorph to accommodate the large thermal motion of the exocyclic groups. In principle, such contributions to entropic stabilization can be addressed quantitatively based on a calculation of vibrational modes. Unfortunately, the computational cost of this calculation is at present prohibitive.

We may also address the greater stability of the triclinic polymorph via a different route by comparing the crystal structures of the monohydrates of cholesterol and stigmasterol and their thermal characteristics. As already discussed, stigmasterol, even in its early stage of formation of a trilayer on water, forms a monoclinic *p*2_1_ structure analogous to the monoclinic *A*2 form of cholesterol. On further growth, stigmasterol retains its original 10 × 7.5 Å^2^ unit cell and space group, unlike the cholesterol monohydrate, which undergoes a transformation to a triclinic *P*1 cell. The atomic displacement parameters (ADPs) of the exocyclic group of stigmasterol appear to be about twice as large as those of the rigid steroid fragment, according to Figure 6b in ref (31). We suggest that the exocyclic moiety of cholesterol is more flexible than the corresponding group of stigmasterol that, as mentioned above, contains a rigid >C=C< system in order to pack effectively in the triclinic unit cell.

Importantly, 2D crystals of cholesterol bilayers, hydrated on both sides and segregating from mixed bilayers with phospholipids, always appear in the monoclinic polymorph.^14−17^ The monoclinic form segregated from mixed bilayers with phospholipids eventually transforms into the triclinic polymorph.^17^ The rate of transformation between the polymorphs depends on the phospholipid environment.^23,45^ Thus, as an example, only macroscopic 3D triclinic crystals are observed associated with sphingomyelin-containing mixed bilayers, whereas initial monoclinic crystals are retained and developed when they are formed from DPPC-containing mixed bilayers.^18,23^ Monoclinic helical crystals formed from solutions with bile acids are also relatively long-lived before transformation to the more stable triclinic polymorph.^19^ Helical triclinic crystals of cholesterol have also been characterized by synchrotron X-ray diffraction, yielding unit cell dimensions similar to that of the thermodynamically stable polymorph of cholesterol·H_2_O but with a *c* axis three times as long.^46^ In the above systems, the lipid environment determines the rate of transformation. Therefore, while cholesterol crystals found in atherosclerotic plaques were exclusively identified as the triclinic polymorph,^5,47^ it stands to reason that they could have formed as monoclinic crystals, having had years to transform prior to extraction. In this respect, we note that a model has been presented above for the induction of the monoclinic form via an epitaxial fit to a bilayer of cholesteryl palmitate, a molecule present in atherosclerotic lesions. The abundance of needle-like crystals in mature plaques is tantalizing in suggesting that this needle-like morphology may be a vestige of the initial formation of crystals in the monoclinic polymorph. This conclusion is further supported by the observation of monoclinic helical crystals in freshly fixed macrophage cells (the cholesterol scavengers in atherosclerosis) to which cholesterol was administered in excess.^23^

## Conclusions

In conclusion, we presented a comprehensive computational study of the two crystal polymorphs of cholesterol·H_2_O, with an emphasis on the lesser-known monoclinic one. Using first-principles calculations based on the dispersion-augmented DFT, we confirmed the known features of the experimentally determined structures. Furthermore, we refined the structure of the monoclinic polymorph by obtaining a fully extended H-bonded network comprising the sterol hydroxyl groups and water molecules, in an arrangement akin to that of hexagonal ice. We further suggested that this network may exist in related structures, notably that of cholestanol·2H_2_O. The ice-like H-bonded network is found in the crystal structure of hydrated stigmasterol as a grown crystal and, in all probability, as a nucleus composed of three layers. The total energy of the newly refined monoclinic form of cholesterol·H_2_O was found to be similar to that of the triclinic one, suggesting that kinetic and environmental effects may play an important role in determining the polymorphic nucleation of cholesterol·H_2_O. We have also invoked the crystalline and thermal properties of stigmasterol hydrate to help rationalize the polymorphic and thermal properties of cholesterol·H_2_O. We have been able to rationalize a single crystal to single-crystal symmetry transformation of the monoclinic form of cholesterol·H_2_O on increased interlayer growth from one to several cholesterol bilayers. We have also discussed how our findings lend support to and rationalize the observation of nucleation of the monoclinic structure of cholesterol in hydrated lipid membranes, followed by transformation to the triclinic counterpart. Finally, we have found an epitaxial match between the cholesteryl surface of a single bilayer of the ester cholesteryl palmitate, which is found in atherosclerotic lesions, and the corresponding surface of monoclinic cholesterol·H_2_O, leading to its proposed nucleation.

## Methods

### Structure Construction

The crystal structure of the monoclinic cholesterol monohydrate was taken from Solomonov et al.,^13^ with hydrogen atoms added using Materials Studio 6.1.^48^ The unit cell was then transformed to a primitive cell, containing half the atoms, with a single water-hydroxyl layer. We derived the primitive cell of monoclinic cholesterol using the phonopy package,^49^ which determines the transformation matrix from the input unit cell to the primitive one, *M*_p_. For the monoclinic *A*2 unit cell, *M*_p_ based on the *A*2 conventional unit cell is given by:
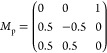
These values agree with those displayed in Table 5.1 of the International Tables for Crystallography.^50^ The primitive cell of cholestanol dihydrate was derived in a similar matter using the same transformation matrix.

For vacuum calculations of the *p*2_1_ and *p*2 structures and the composite crystal, a layer of vacuum of ∼10 Å was added to terminate the periodicity in the *c*-axis. To study the contribution of inter- and intra-molecular forces on the molecular packing of cholesterol monohydrate crystals, isolated single cholesterol molecules of the triclinic and monoclinic polymorphs had a vacuum spacing of at least 10 Å in all three axes. The same vacuum spacing was also applied to the isolated infinite H-bonded networks, with the cholesterol C atoms connected to the cholesterol hydroxyl (OH) groups replaced with H.

Molecular structures were visualized using VESTA, a three-dimensional visualization system for electronic and structural analysis.^51^

### DFT Calculations

Electronic structures, total energies, and geometries were calculated by solving the Kohn–Sham equations of DFT within the generalized gradient approximation (GGA), using the Perdew–Burke–Ernzerhof (PBE) exchange–correlation functional.^52^ The total energy was augmented by Tkatchenko–Scheffler van der Waals (TS-vdW) pair-wise dispersive terms.^53^ Most of the calculations were carried out using version 5.4.4 of the Vienna *ab initio* simulation package (VASP)^54^ plane-wave basis code,^55,56^ where ionic cores are described by the projected augmented wave (PAW) method.^54,57^ A plane wave energy cutoff of 920 eV was used in all calculations. Further calculations of the composite crystal, as well as MBD^27^ and MBD-NL^28^ based calculations, were performed using the Fritz Haber Institute *ab initio* molecular simulations (FHI-aims) package.^58^ FHI-aims is an all-electron, full-potential electronic structure code utilizing numeric atom-centered basis functions for its electronic structure calculations, which we used to speed up testing and address large system computations. We employed the “tight” settings, in which the *tier 2* basis set is used for the light elements 1–10. It is considered to result in converged conformational energy differences at a level of a few meV.^58^ The Brillouin zone was sampled using a Gamma-centered Monkhorst–Pack *k*-point grid^59^ of 3 × 3 × 1 and 2 × 2 × 7 for the triclinic and monoclinic structures, respectively, and 2 × 2 × 8 for cholestanol dihydrate. With these numerical choices, structural parameters were found to be numerically converged to 0.01 Å. Total energies were converged to <1 meV/atom. Atomic forces in the system were relaxed to 10^–4^ eV/Å and stress was relaxed to 10^–3^ kB.

## Supporting Information Available

Details of crystal structure determination of monoclinic cholesterol monohydrate; eight H-bonding motifs of the triclinic cholesterol·H_2_O polymorph; generation of a H-bonding network in the monoclinic crystal structure of cholesterol·H_2_O; four H-bonding motifs of the monoclinic cholesterol·H_2_O polymorph; model structures used to study the contribution of intra-molecular forces on the molecular packing of cholesterol·H_2_O crystals; structural parameters of the monoclinic cholesterol·H_2_O optimized by DFT, compared to the experiment; temperature dependence of the *d*-spacing of monoclinic cholesterol·H_2_O; morphologies of the triclinic and monoclinic crystals of cholesterol·H_2_O; hypothetical *P*2_1_ crystal structures of cholesterol·H_2_O: structure and energy profiles; atomic center of mass coordinates and the vector distance between the centers of mass of the atoms of the exocyclic moieties of molecules *A* and *B* of the monoclinic cholesterol·H_2_O; single cholesterol bilayers in which two leaflets are related by a twofold screw axis *p*2_1_, as opposed to twofold axes *p*2 and *p*1; model for the packing arrangement of the monoclinic cholestanol·2H_2_O; packing arrangement of the stigmasterol·H_2_O; GIXD patterns and Bragg rods of the stigmasterol hydrate trilayer; and transformation of the monoclinic form on increased interlayer growth (PDF)

## Accession Codes

CCDC 2157904–2157917 contain the supplementary crystallographic data for this paper. These data can be obtained free of charge via www.ccdc.cam.ac.uk/data_request/cif, or by emailing data_request@ccdc.cam.ac.uk, or by contacting The Cambridge Crystallographic Data Centre, 12 Union Road, Cambridge CB2 1EZ, UK; fax: +44 1223 336033.

## Footnotes

a We note that following the relaxation process, the system was first trapped in a local minimum, which was overcome by reorienting one H-atom to fit the model in Figure S3. The local minimum configuration is ∼1.5 kcal/mol per molecule higher in energy compared to the global minimum configuration.

b Similarly, the difference in energy of the isolated, infinite water layer (see Methods for details) is ∼0.5 kcal/mol per molecule, with the arrangement of the triclinic form being slightly more stable.
