# Supplementary material for: Polymorphism, Structure, and Nucleation of Cholesterol·H2O at Aqueous Interfaces and in Pathological Media: Revisited from a Computational Perspective
Source: J Am Chem Soc. 2022 Mar 16;144(12):5304–14. doi: 10.1021/jacs.1c10563 (PMC8972249; doi:10.1021/jacs.1c10563)
Supplement: Supplementary file 1 — ja1c10563_si_001.pdf [file ja1c10563_si_001.pdf]

Supporting Information

Polymorphism, Structure, and Nucleation of Cholesterol.H_2O at Aqueous Interfaces and in Pathological Media: Revisited from a Computational Perspective

Margarita Shepelenko,[a] Anna Hirsch,[a] Neta Varsano,[b] Fabio Beghi,[c]
Lia Addadi,[b] Leeor Kronik,*[a] and Leslie Leiserowitz*[a]

[a] Department of Molecular Chemistry and Materials Science, Weizmann Institute of Science, Rehovoth 7610001, Israel
[b] Department of Structural Biology, Weizmann Institute of Science, Rehovoth 7610001, Israel
[c] Department of Chemistry, Università Degli Studi Di Milano, I-20122 Milano, Italy
*To whom correspondence should be addressed. Email: Leeor.Kronik@weizmann.ac.il, Leslie.Leiserowitz@weizmann.ac.il

§1. Details of crystal structure determination of monoclinic cholesterol monohydrate.

As mentioned in the main text, the three-dimensional structure of the monoclinic polymorph was determined based on only three cholesterol bilayers. Structure elucidation of a thin molecular layered system at the air-water interface is generally not a straightforward process for complex systems, requiring a multi-pronged approach. Such thin films can be characterized to near-atomic resolution by synchrotron grazing incidence x-ray diffraction (GIXD), a method that has been applied to study molecular self-assembly of crystalline films ranging from one to several layers thick, such as amphiphiles, and thin film crystallites of supramolecular architecture.(1, 2) Cholesterol monoclinic films composed of 1-3 bilayers, azimuthally misoriented on the water surface,(3) were amenable to detailed structural characterization, via GIXD, because the diffraction peaks were by and large sharp and intense, as a result of pronounced molecular ordering. Thus, each of these crystalline films yielded well-defined {*h,k*} Bragg 'rods', (Figure S1.1) which were indexed in terms of a two-dimensional (2D) rectangular 10×7.5 Å^2 unit cell, parallel to the plane of the water surface. The single bilayer is of symmetry $p2_1$, namely the two leaflets are related by twofold screw symmetry. A film of three bilayers yielded intensity maxima along the Bragg rods, which were sufficiently intense, sharp, and well-separated to be regarded as regular {*hkl*} reflections of a 3D crystal (with unit cell dimensions of a = 10.15(2), b = 7.57(2), c = 68.2(3) Å, β = 94.8(5)°).(3)

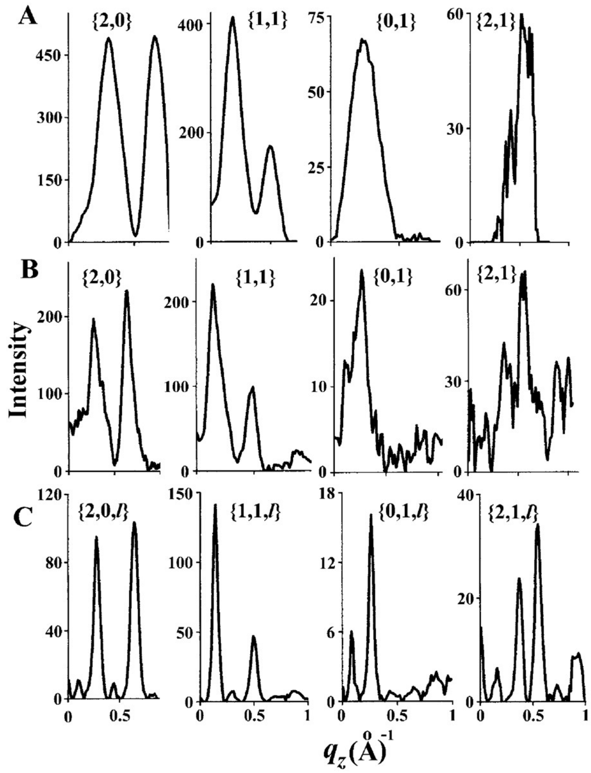

Fig. S1.1. Bragg rods of three different compressed films at the air-water interface of cholesterol with the following thicknesses. (A) Single cholesterol bilayer . (B) Two cholesterol bilayers. (C) Three cholesterol bilayers. Reprinted from our previous studies, ref. (3). Copyright: 2005, *Biophysical Journal.*

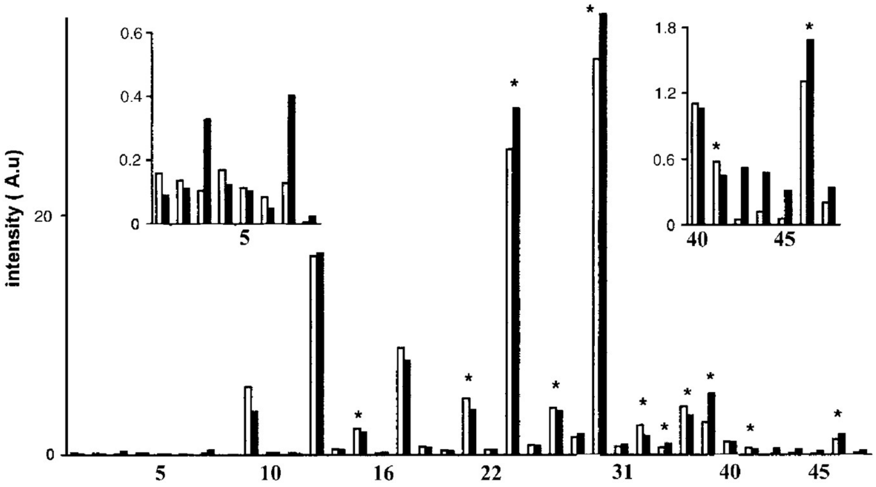

Fig. S1.2. Observed (*solid bars*) and calculated (*open bars*) values of the 48 x-ray structure factors $|F(h,k,l)|^2$, the latter based on the structural model of the monoclinic *A*2 phase of cholesterol.H_2O. The $|F(h,k,l)|^2$ values are displayed as a function of increasing $q(h,k,l)$, = $2\pi d^*(h,k,l)$, equally separated for clarity of presentation. The bars marked with an asterisk, represent reflection pairs of the type $(2,k,l)$ and $(-2,k,l + 2)$, which are heavily overlapped in the GIXD pattern and thus are shown superimposed. Reprinted from our previous studies, ref. (3). Copyright: 2005, *Biophysical Journal.*

The symmetry of this crystal structure of monoclinic monohydrate was assigned based on 48 reflections, the *hkl* indices of which obeyed the condition $k + l = 2n$. The cholesterol molecular packing was then generated by utilizing the cholesteryl myristate crystal structure[(4)] as an initial model. This is because the cholesteryl myristate crystal structure exhibits a bilayer motif with *a,b* axial dimensions and a β angle, very similar to those of monoclinic cholesterol.H_2O and because its 32.9 Å bilayer thickness almost matches half the length of the *c*-axis of the monoclinic cholesterol (assuming the latter is a monohydrate phase with a 1.5 Å thick water layer). This was consistent with the calculated density of the monoclinic form (1.029 g/mL) which almost matched the density of the triclinic form of cholesterol (1.048 g/mL).

Even with this structural information some ambiguities still had to be resolved. Firstly, the cholesterol bilayer was generated via a twofold (2) axis as in the cholesteryl myristate crystal structure, with space group *A*2. However, because this monoclinic space group incorporates rows of twofold (2) and twofold screw (2_1) axes parallel to *b* and alternating along the *c* axis, the cholesterol bilayer could have been constructed across the 2_1 axes instead. Indeed, the latter arrangement occurs in the organization of a single cholesterol bilayer on the air-water interface,[(5)] and in the crystal structures of the tridecanoate and stearate derivatives of cholesterol.[(6)] The model crystal structure, constructed with a cholesterol bilayer whose two leaflets were related by twofold symmetry, was refined by least squares X-ray structure factor analysis. In this procedure, the two sterol molecules per asymmetric unit were treated as rigid bodles (corresponding to 11 parameters), which yielded a satisfactory fit between the observed and computed X-ray structure factors shown in Figure S1.2. The reliability index, $R = \sum \left| F^2_{o(hkl)} - F^2_{c(hkl)} \right| / \sum F^2_{o(hkl)}$, where $F_{(hkl)}$ is the X-ray structure factor of a particular diffraction peak and 'o' and 'c' refer to its observed and calculated values, proved to be as low as 13.5%, indicating an overall correct structure.

§2. Eight H-bonding motifs of the triclinic cholesterol.H2O polymorph.

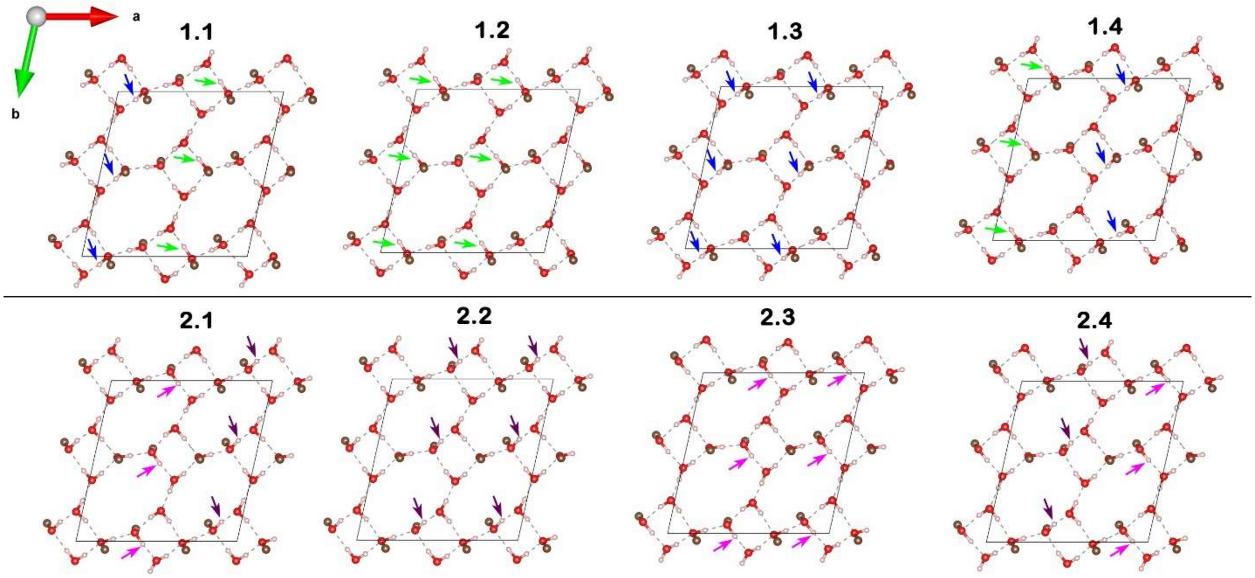

Fig. S2. Eight H-bonding motifs of the triclinic cholesterol.H_2O polymorph. The number before each dot refers to the configuration of the cholesterol molecules connected by hydrogen bonds between the two hydroxyl groups. In 1.1 to 1.4, one of the oxygens acts as a donor and the other one as an acceptor. In motifs 2.1 to 2.4, cholesterol oxygen molecules switch their roles. The number after the dot refers to the different orientations of the acceptor hydrogen atom, as shown with colored arrows. The donor hydrogen bonding orientation remains unchanged within each donor-acceptor configuration. 1.3 corresponds to the motif originally determined by Craven,[7, 8] based on computational refinement of this structure by Frincu et al.[9]

Table S2.1. Optimized unit cell parameters (in Å, degrees and $Å^3$) for the proposed H-bonding structural motifs of the triclinic polymorph shown in Fig. S2 at PBE-TS level of theory.

motif	a	b	c	α	β	γ	V
**1.1**	11.85	11.95	34.16	92.1	98.8	102.0	4663.3
**1.2**	11.86	11.94	34.17	91.9	98.8	102.0	4669.0
**1.3**	11.84	11.96	34.15	92.3	98.8	101.9	4662.9
**1.4**	11.85	11.96	34.17	92.1	98.8	101.9	4668.9
**2.1**	11.85	11.94	34.18	92.0	98.9	102.0	4663.4
**2.2**	11.84	11.95	34.17	92.2	98.8	101.9	4662.9
**2.3**	11.88	11.93	34.19	91.8	98.9	102.0	4671.1
**2.4**	11.87	11.94	34.18	91.9	98.8	102.0	4670.3

Table S2.2. Optimized average O···O bond lengths in Å for the proposed H-bonding structural motifs of the triclinic polymorph at PBE-TS level of theory. The different H-bonded rings in Fig. $2C_1$ are labeled in grey by r_i and R_i, which refer to tetragons and octagons, respectively; subscript i from 1 to 4 designates the unique polygons of each type.

motif	R_1	R_2	R_3	R_4	r_1	r_2	r_3	r_4
1st	2.75 ± 0.07	2.73 ± 0.09	2.77 ± 0.08	2.76 ± 0.10	2.82 ± 0.13	2.77 ±0.11	2.80 ± 0.11	2.75 ± 0.09
2nd	2.74 ± 0.09	2.72 ± 0.10	2.79 ± 0.10	2.76 ± 0.10	2.83 ± 0.15	2.76 ± 0.12	2.81 ± 0.11	2.75 ± 0.11
3rd	2.74 ± 0.08	2.72 ± 0.08	2.78 ± 0.06	2.77 ± 0.11	2.81 ± 0.09	2.76 ± 0.11	2.81 ± 0.14	2.74 ± 0.11
4th	2.73 ± 0.08	2.72 ± 0.09	2.78 ± 0.07	2.78 ± 0.10	2.80 ± 0.09	2.76 ± 0.09	2.81 ± 0.14	2.77 ± 0.13
5th	2.73 ± 0.06	2.74 ± 0.10	2.78 ± 0.08	2.77 ± 0.09	2.82 ± 0.13	2.76 ± 0.09	2.80 ± 0.11	2.77 ± 0.13
6th	2.74 ± 0.06	2.73 ± 0.10	2.79 ± 0.10	2.77 ± 0.09	2.83 ± 0.15	2.76 ± 0.09	2.81 ± 0.11	2.75 ± 0.10
7th	2.75 ± 0.06	2.73 ± 0.07	2.79 ± 0.10	2.77 ± 0.09	2.82 ± 0.12	2.77 ± 0.09	2.80 ± 0.11	2.76 ± 0.10
8th	2.75 ± 0.09	2.72 ± 0.06	2.79 ± 0.10	2.76 ± 0.09	2.82 ± 0.12	2.77 ± 0.13	2.80 ± 0.11	2.76 ± 0.11

Table S2.3. Computed total energies for the proposed H-bonding structural motifs of the triclinic polymorph shown in Fig. S2 relative to the lowest energy motif, at PBE-TS level of theory.

motif	1.1	1.2	1.3	1.4	2.1	2.2	2.3	2.4
Δ, [kcal/mol]/molecule	0.00	0.02	0.06	0.08	0.06	0.13	0.12	0.20

§3. Generation of H-bonding network in the monoclinic crystal structure of cholesterol.H_2O. Each water molecule in the crystal structure of hexagonal ice participates in four H-bonds in a three-dimensional tetrahedral arrangement, incorporating two O-H proton donor bonds and two proton acceptor lone-pair electron lobes. The C-OH groups of, e.g. methanol molecules, each participates in two H-bonds forming a one-dimensional H-bonded network, incorporating one OH proton donor, which is linked to a proton acceptor O atom via its lone pair electron lobes. Hence, we may expect ideally that in the crystal structure of monoclinic cholesterol.H_2O, a two-dimensional H-bonded layer network of sterol C-OH groups and H_2O molecules would interlink such that each O atom forms three O-H…O bonds as in the crystal structure of the triclinic polymorph of cholesterol.H_2O and in the (002) bilayer arrangement of hexagonal ice. Each sterol O atom would donate one proton and accept two protons, whereas each water molecule would do the reverse, i.e., donate two protons and accept one proton. Thus, given the cholesterol:water molar ratio of 1:1, each water O atom would be bonded to two sterol O atoms and one water O atom, and each sterol O atom would be H-bonded to two water O atoms and one sterol O atom. Since the cholesterol molecules in each layer are too bulky to easily form H-bonds with each other, the H-bond between the two sterol C-OH groups must interlink the molecular layers across the twofold screw axes. This is indeed true for the starting model structure of the monoclinic form (Fig. S3). Given meagre information as to the C-O sterol bilayer arrangement in the monoclinic cholesterol, it is still possible to construct a feasible H-bond motif, which satisfies the above conditions, as depicted in Fig. S3. In this figure, we have drawn circles of a 3 Å radius about the atomic centers of the sterol O atoms, to generate the possible positions of the water O atoms.

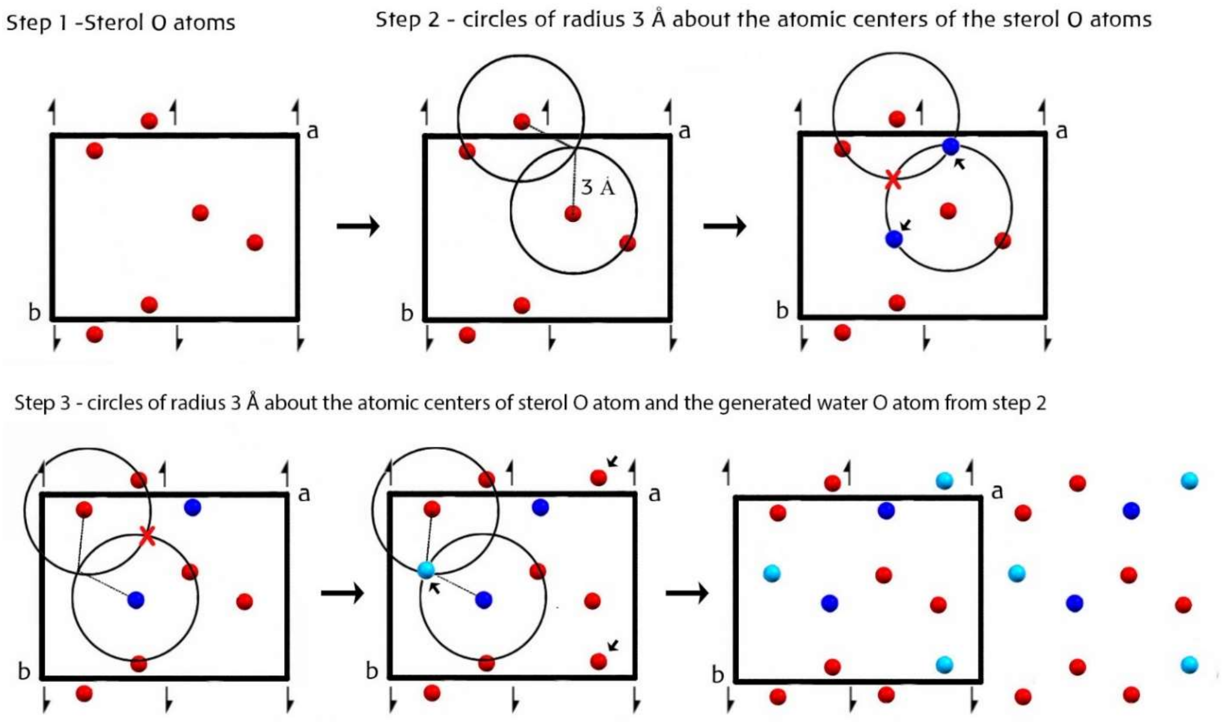

Fig. S3. Various steps **(1-3)** in the generation of the H-bonded *ab* bilayer in monoclinic cholesterol.H_2O. **(1)** Apply the twofold screw elements shown to generate the symmetry-related positions of the two asymmetric sterol O atoms (in red), which belong to the opposite leaflet of the bilayer. Note that the neighboring sterol O atoms, belonging to opposite leaflets, are separated by ~ 3 Å and so H-bonded to each other. **(2)** Draw circles of radius 3 Å about the centers of two symmetry-related sterol O atoms as shown. These two circles intersect at two positions. The position labeled with a red × cannot accommodate a water O atom, because it would form a close-packed isosceles triangle with two sterol O atoms. The other position of intersection is occupied by a water O(1) atom (blue), which clearly can form H-bonds with two neighboring sterol O atoms. Now generate the position of the symmetry related water O(1) atom, marked by an arrow. **(3)** Draw circles of radius 3 Å about the atomic centers of sterol O atom and the generated water O(1) atom from step 2 as shown. These two circles intersect at two positions. The position labeled with a red × cannot accommodate a water O atom, because it would form a close-packed isosceles triangle with two sterol O atoms. The other position of intersection is occupied by a water O(2) atom (light blue), which clearly can form H-bonds with two neighboring sterol O atoms.

§4. Four H-bonding motifs of monoclinic cholesterol.H_2O polymorph.

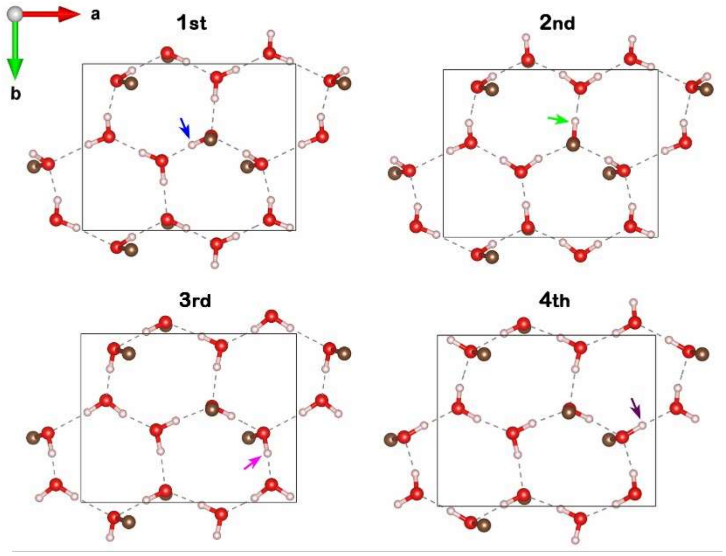

Fig. S4. Four H-bonding motifs of the monoclinic cholesterol.H_2O polymorph. In motifs 1 and 2, one of the sterol O atoms acts as a donor and the other one as an acceptor, and in motifs 3 and 4 their roles are reversed. The H_2O molecules adapt to these configurations, yielding a maximum of 4 H-bonding motifs.

Table S4.1. Computed total energies for the generated structural motifs of the monoclinic polymorph, relative to the first motif, as shown in Fig. S4, at PBE-TS level of theory.

motif	1st	2nd	3rd	4th
Δ, [kcal/mol]/molecule	0	0.18	0.23	0.67

Table S4.2. Optimized unit cell parameters (in Å, degrees and $Å^3$) and corresponding averaged H-bonding OH···O distances in the two hexagonal rings, R_1 and R_2, for all generated H-bonding motifs of the monoclinic cholesterol.H_2O polymorph at PBE-TS level of theory.

motif	*a*	*b*	*c*	β	V = *abc*·sinβ	$R_{O\text{-}O}$ (R_1)	$R_{O\text{-}O}$ (R_2)
1st	9.63	7.46	66.91	96.3	4778.7	2.74 ± 0.10	2.87 ± 0.11
2nd	9.62	7.46	67.01	96.2	4781.7	2.76 ± 0.08	2.85 ± 0.16
3rd	9.63	7.47	66.78	96.3	4773.7	2.78 ± 0.07	2.83 ± 0.13
4th	9.62	7.49	66.66	96.0	4772.3	2.83 ± 0.03	2.77 ± 0.07

§5. Model structures used to study the contribution of intra-molecular forces on the molecular packing of cholesterol.H_2O crystals.

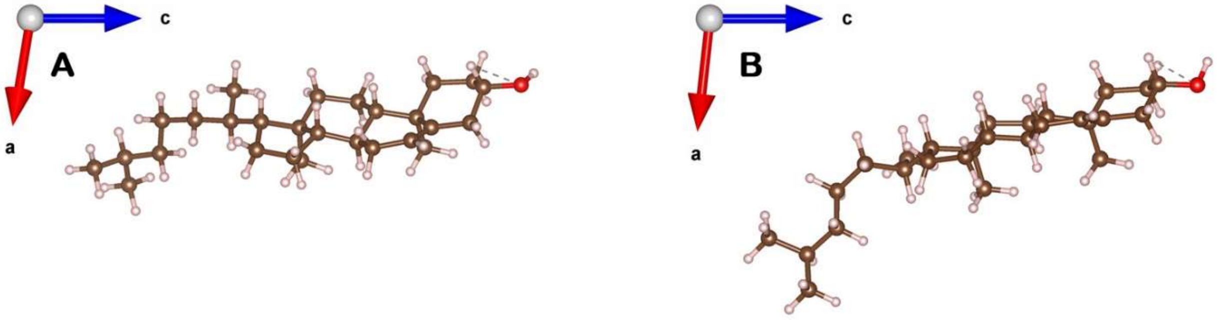

Fig. S5. Model structures used to study the contribution of intra-molecular forces on the molecular packing of cholesterol.H_2O crystals: Isolated single cholesterol molecule for the triclinic **(A)** and monoclinic **(B)** polymorphs.

§6. Structural parameters of the monoclinic cholesterol.H_2O optimized by DFT compared to experiment.

Table S6. Optimized structural parameters (in Å, degrees and $Å^3$) for the lowest energy H-bonding motif of the monoclinic polymorph at the PBE-TS, MBD and MBD-NL levels of theory, compared to the experimental data of Solomonov et al.[(3)]

method	a	b	c	α	β	γ	V
experiment	10.15	7.57	68.20	90.0	94.8	90.0	5222.0
TS	9.66	7.48	66.99	90.0	96.4	90.0	4809.4
MBD	9.51	7.62	66.02	90.0	96.7	90.0	4749.2
MBD-NL	9.70	7.51	67.89	90.0	100.6	90.0	4861.5
% TS	4.82	1.22	1.78	0.0	1.6	0.0	7.90
% MBD	6.30	0.61	3.19	0.0	2.1	0.0	9.05
% MBD-NL	4.47	0.74	0.46	0.00	6.12	0.00	6.90

§7. Temperature dependence of the *d*-spacing of monoclinic cholesterol.H_2O.

Table S7. Temperature dependence of the *d*-spacing of monoclinic cholesterol.H_2O, measured by electron diffraction (ED) and grazing incidence X-ray diffraction (GIXD), and calculated by DFT at the PBE-TS level of theory.

method	T, [K]	d_{200} [Å]	d_{111} [Å]
TS	0	4.8	5.84
Cryo ED[10]	90	4.9	5.8
GIXD[3]	278	5.06	6.01
Δ in % , TS vs cryo ED		2.04	-0.69
Δ in % , TS vs GIXD		5.14	2.83

§8. Morphologies of the triclinic and monoclinic crystals of cholesterol.H_2O.

Theoretical morphology simulations

Theoretical crystal morphologies were obtained using the Materials Studio Morphology module 6.1.[11] The crystal shape was simulated by use of the "growth morphology" approach. Attachment energies and surface energies were calculated using the Dreiding force field.[12] We note, however, that the morphology simulation method does not take into account solvent effects and possible surface reconstructions, which could have a profound influence on experimentally observed morphologies.

Cholesterol crystals were grown on supported lipid bilayers (Fig. S8C and F_1) following a procedure described in detail in our previous work.[13] The procedure for crystal growth from cell culture models (Fig. S8F_2), following crystal characterization using cryo-transmission electron diffraction and cryo–soft X-ray tomography, is described in Varsano et al.[14]

The theoretical growth morphologies of both the triclinic and monoclinic structures were determined using interatomic potential energy computations (see Methods section for details), which although qualitative, yielded results which by and large match the observed morphologies of the crystals grown in solution. The computational analysis predicts a crystal plate habit with a rhomb-like shape (Fig. S8), expressing a dominant (001) face, which corresponds to the plane parallel to the bilayer. All expressed faces correspond to low index flat planes. The crystal plate is delimited by the {100}, {010}, and {011} side faces, where the rhomb shape reflects the close similarity between the unit cell dimensions and the molecular interactions in the *a* and *b* directions. Two cut-off edges expose minor $\{1\bar{1}0\}$ and $\{1\bar{1}\bar{1}\}$ side faces. This is in good agreement with the

experimental morphology of triclinic cholesterol crystals grown from water solutions, which appear as thin quadrilateral plates (Fig. S8B, C), where a bi-axial growth along *a* and *b*, forming an angle of 101°, is found. The crystals are so thin that accurate determination of the side faces is difficult to perform.

For the monoclinic structure, the growth analysis predicts a crystal habit with a rectangular shape (Fig. S8D), expressing the hydrogen-bonded (001) layer as a dominant plate face. The crystals elongate along the *b* direction and are delimited by {102} and {100} side faces along the *b*-axis and {011} side faces along the *a*-axis. Experimental information as to the facets exhibited by the 3D monoclinic polymorph is lacking. We only managed to examine 3D faceted monoclinic crystals when we nucleated cholesterol on supported mixed lipid bilayers with saturated phospholipids. Representative results for monoclinic crystals thus grown (Fig. S8E,F) show similarities to the theoretical prediction in the tendency to elongate along *b* (see model Fig. S8E). The very thin crystals develop diagonal end faces ($11l$) and ($\bar{1}1l$) with an angle between them of ~106°, rather than (010) end faces (Fig. S8E,F_1). The long aspect ratio results in the monoclinic crystals having a needle shape (Fig. S8E,F_1). Crystals grown from supported bilayers can also form tens of micrometer-long ribbons elongated in *b* (Fig. S8F_2). It is noteworthy that the habit of cholestanol crystals reported by D. Hodgkin (Fig. 5D and S13) matches the transmission electron microscope image of a monoclinic crystal grown on a supported lipid bilayer (Fig. S8F_1).

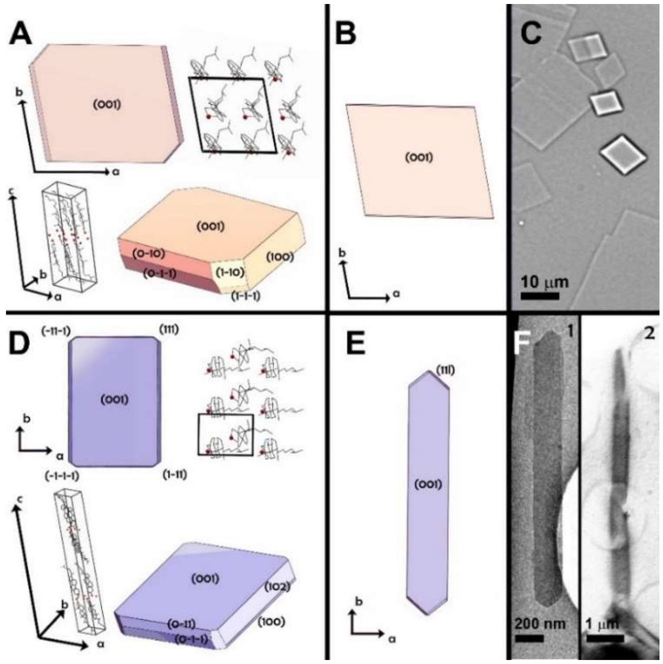

Fig. S8. **(A)** and **(D)**: Theoretical equilibrium growth morphology of the triclinic (panel **A**) and monoclinic (panel **D**) structures. The corresponding molecular packing arrangement for each crystal orientation is presented accordingly. **(B)** and **(E)**: Model representation of the experimental morphology of triclinic **(B)** and monoclinic **(E)** crystals. **(C)**: Optical images of triclinic crystals grown on supported lipid bilayer. **(F_1)**: Transmission electron microscope image of a monoclinic crystal grown on a supported lipid bilayer; **(F_2)**: Helical crystal of the monoclinic polymorph grown from a macrophage cell under conditions of cholesterol supersaturation. The image is a reconstructed segmented volume from a cryo-soft X-ray tomogram.[14]

§9. Hypothetical $P2_1$ crystal structures of cholesterol.H_2O: structure and energy profile.

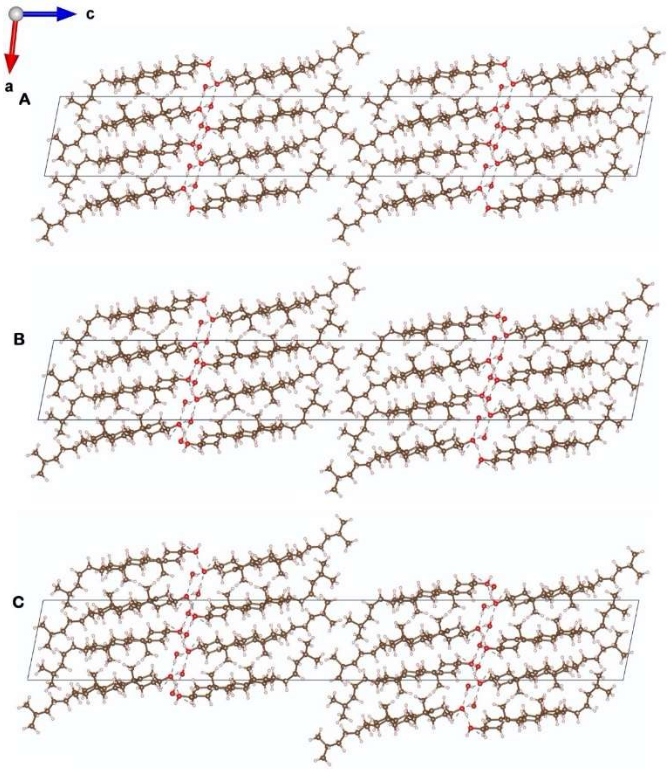

Fig. S9.1. A series of hypothetical $P2_1$ crystal structures of cholesterol.H_2O, viewed along the *b*-axis, generated by replacing the twofold axes of the *A*2 polymorph by twofold screw axes and subsequent **(A)** zero, **(B)** 0.1*a* and **(C)** 0.2*a* offsets of the adjacent cholesterol bilayers along the *a*-axis, at the hydrophobic interface. The hydrophilic interface was kept fixed as in the *A*2 crystal structure, thus maintaining the original H-bonded bilayer system across which the corresponding cholesterol layers are related by twofold screw symmetry.

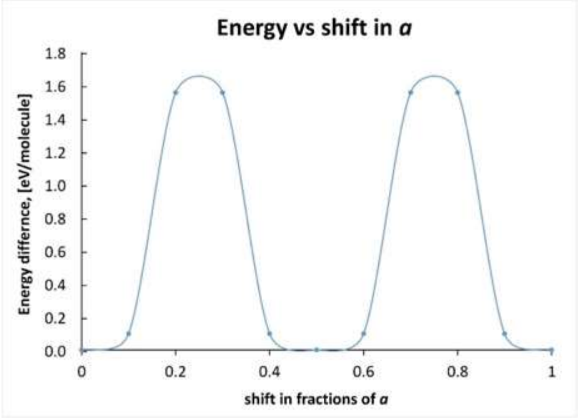

Fig. S9.2. DFT-computed, static energy profile of a series of hypothetical $P2_1$ crystal structures of cholesterol.H_2O, generated by replacing the twofold axes of the *A*2 polymorph by twofold screw axes and subsequent offset of the adjacent cholesterol bilayers along the a-axis, at the hydrophobic interface. The hydrophilic interface remained the same as in *A*2 crystal structure, thus maintaining the original H-bonded bilayer system across which the corresponding cholesterol layers are related by twofold screw symmetry.

§10. Atomic/center of mass coordinates and the vector distance between the centers of mass of the atoms of the exocyclic moieties of molecules *A* and *B* of the monoclinic cholesterol.H_2O.

Table S10.1. Atomic and center of mass coordinates of the (terminal) atoms of the exocyclic moieties of molecules *A* and *B* of the monoclinic cholesterol.H_2O. Each row contains atoms to be added to the atoms of the previous row for calculation of the center of mass (3 terminal methyl groups, 5 terminal methyl groups and full exocyclic chain, as shown in the figures for each row). The center_of_mass_*i*, was estimated as an average of the atomic coordinates, divided by the number of atoms. Center of mass index corresponds to the number of the terminal methyl groups.

mol *A*				mol *B*			
**atom**	***x***	***y***	***z***	**atom**	***x***	***y***	***z***
C51	0.71254	0.18859	0.03959	C24	1.15371	0.59873	0.0481
H57	0.78792	0.20971	0.05299	H88	1.13583	0.45325	0.04611
H58	0.68147	0.3243	0.03424	H89	1.20508	0.61538	0.06355
C52	0.79097	0.09378	0.02388	C25	1.25647	0.66005	0.03333
H79	0.80044	-0.04885	0.02823	H90	1.28265	0.80207	0.03665
C54 3 methyl groups	0.71198	0.10368	0.00274	C27	1.19413	0.64788	0.01127
H59	0.61147	0.03314	0.002	H94	1.15344	0.51247	0.00763
H60 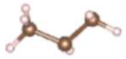	0.77144	0.04353	-0.00864	H95	1.2735	0.67732	0.00111
H61	0.68996	0.24383	-0.00166	H96	1.108	0.74278	0.00766
C50	0.58693	0.08997	0.04658	C23	1.01225	0.69501	0.04599
H55	0.49346	0.1027	0.03559	H86	1.02934	0.84009	0.04818
H56 5 methyl groups	0.61118	-0.05436	0.04766	H87	0.9633	0.68022	0.03043
C53 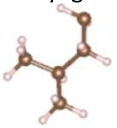	0.93916	0.16971	0.0246	C26	1.39214	0.55134	0.0365
H80	1.00408	0.09098	0.01539	H91	1.46928	0.59994	0.02681
H81	0.9913	0.16914	0.04008	H92	1.37233	0.40901	0.03291
H82	0.9386	0.30904	0.01918	H93	1.44043	0.55816	0.05213
C47	0.44488	0.08022	0.07833	C20	0.9292	0.68972	0.08206
H52	0.34241	0.10444	0.06955	H26	1.04123	0.67579	0.08755
C48 full exocyclic chain	0.46608	-0.12248	0.08056	C21	0.88981	0.88751	0.08297
H76	0.47357	-0.18544	0.06593	H27	0.9156	0.94445	0.09798
H77 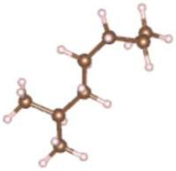	0.38013	-0.18882	0.0871	H28	0.77724	0.90512	0.07879
H78	0.56301	-0.15348	0.09017	H29	0.94437	0.96872	0.07259
C49	0.55936	0.16438	0.06705	C22	0.90835	0.6242	0.06
H53	0.65765	0.15387	0.07712	H30	0.80114	0.66045	0.05373
H54	0.5397	0.309	0.06536	H31	0.91204	0.47709	0.05953
**center_of_mass_3**	0.73	0.13	0.02	**center_of_mass_3**	1.20	0.63	0.03
**center_of_mass_5**	0.76	0.13	0.03	**center_of_mass_5**	1.22	0.63	0.03
**center_of_mass_all**	0.66	0.09	0.04	**center_of_mass_all**	1.10	0.68	0.05

Table S10.2. Vector difference ($\Delta x\mathbf{a} + \Delta y\mathbf{b} + \Delta z\mathbf{c}$ for the ith group) between the centers of mass of the (terminal) atoms of the exocyclic moieties of molecules *A* and *B* of the monoclinic cholesterol.H_2O, estimated as a

difference between the corresponding center of mass coordinates. The index *i* corresponds to the number of (terminal) atoms of each the exocyclic groups of molecules *A* and *B* of the monoclinic cholesterol.H_2O used for calculation.

	x(mol_*B*) - *x*(mol_*A*)	*y*(mol_*B*) - *y*(mol_*A*)	*z*(mol_*B*) - *z*(mol_*A*)
Δ*x*, Δ*y*, Δ*z* of c. of m. 3	**0.47**	**0.50**	**0.01**
Δ*x*, Δ*y*, Δ*z* of c. of m. 5	**0.46**	**0.50**	**0.01**
Δ*x*, Δ*y*, Δ*z* of c. of m. all	**0.44**	**0.59**	**0.00**

§11. Single cholesterol bilayers in which the two leaflets are related by a twofold screw axis $p2_1$, as opposed to a twofold axis *p*2 and *p*1.

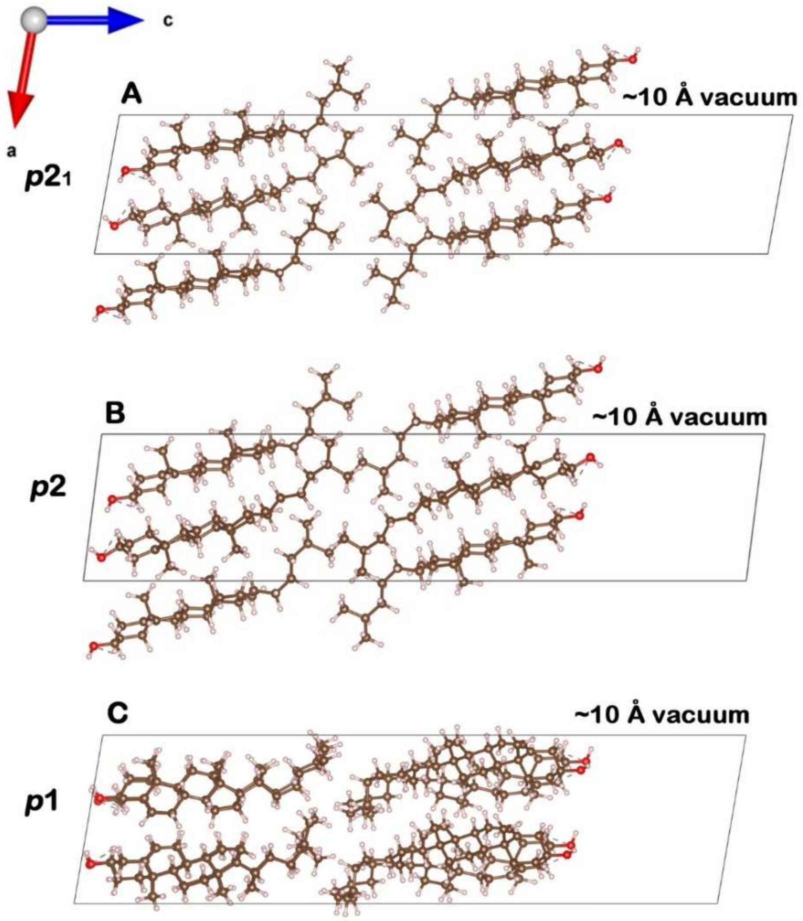

Fig. S11. View along *the* b-axis of single cholesterol bilayers in which the two leaflets are related by a twofold screw axis $p2_1$ **(A)**, as opposed to a twofold axis *p*2 **(B)** and *p*1 **(C)**, followed by a 10 Å vacuum layer.

§12. Model for the packing arrangement of the monoclinic cholestanol.2H_2O.

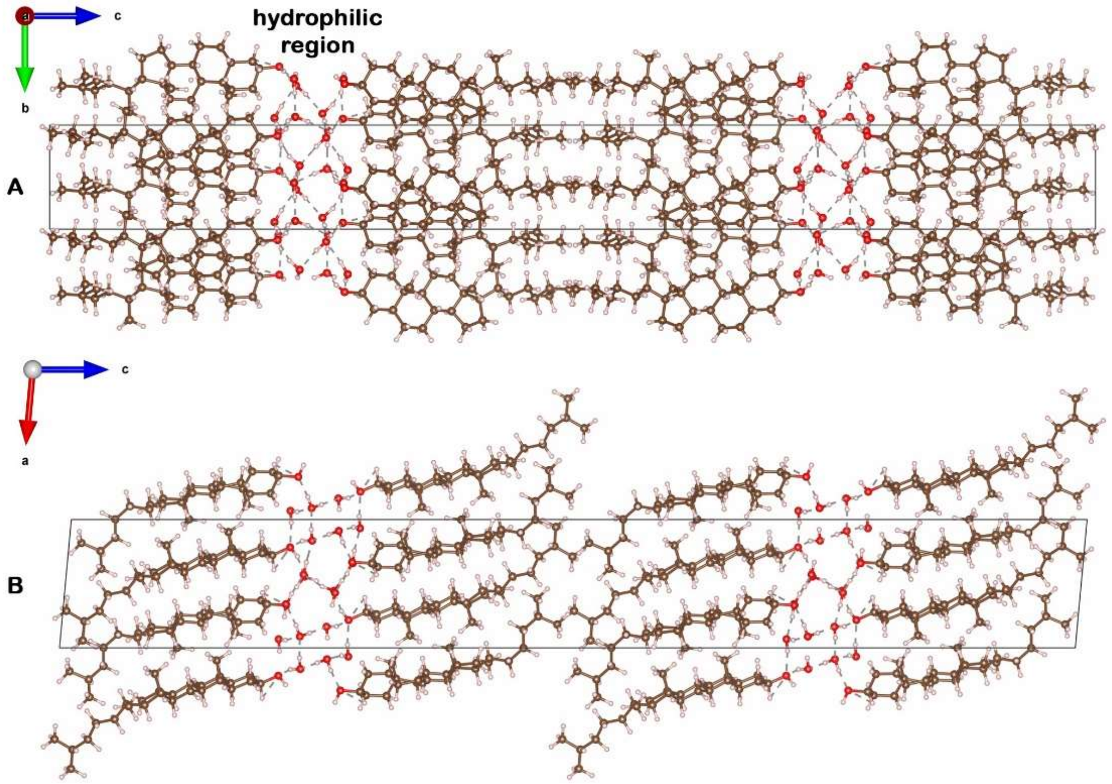

Fig. S12. Model for the packing arrangement of the monoclinic cholestanol.2H_2O unit cell, viewed along the *a*-axis **(A)** and *b*-axis **(B)**. The atoms are color-coded in: white, hydrogen; brown, carbon; red, oxygen. OH···O bonds are represented as grey dashed lines. The unit cell is indicated by a black rectangle.

§13. Packing arrangement of the stigmasterol.H_2O, based on a cif file by Jiang at al. (2001).[(15)]

A

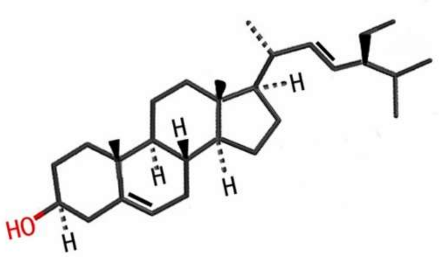

B

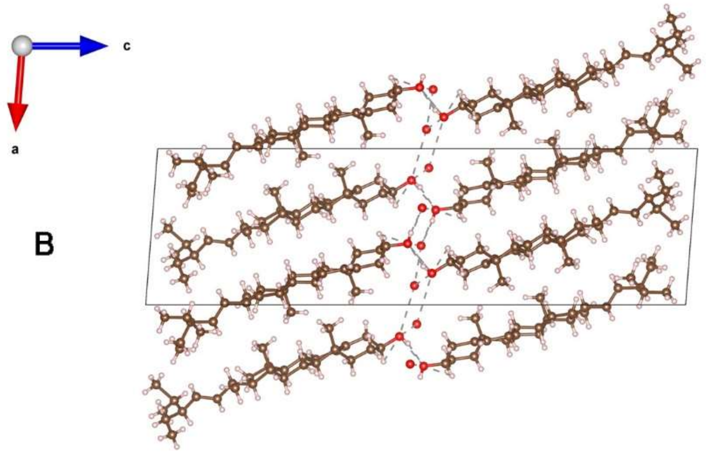

C

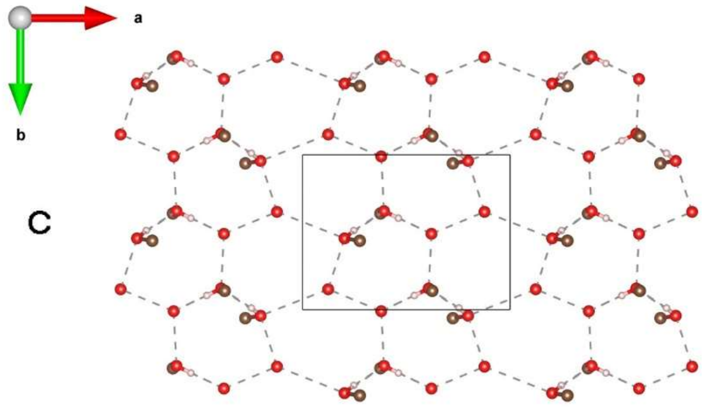

Fig. S13. (A) Stigmasterol molecule. **(B, C)** Packing arrangement of the stigmasterol.H_2O unit cell, viewed along the *b*- and *c*-axes, respectively, based on a cif file by Jiang at al. (2001).[(15)] Water hydrogen atoms are missing from the cif file. Nevertheless, in **(C)** we may recognize an ice-like hydrogen bonding motif as in the monoclinic crystal structure of cholesterol.H_2O. The atoms are color-coded in: white, hydrogen; brown, carbon; red, oxygen. OH···O bonds are represented as grey dashed lines. The unit cell is indicated by a black rectangle.

§14. GIXD patterns and Bragg rods of the stigmasterol hydrate trilayer.

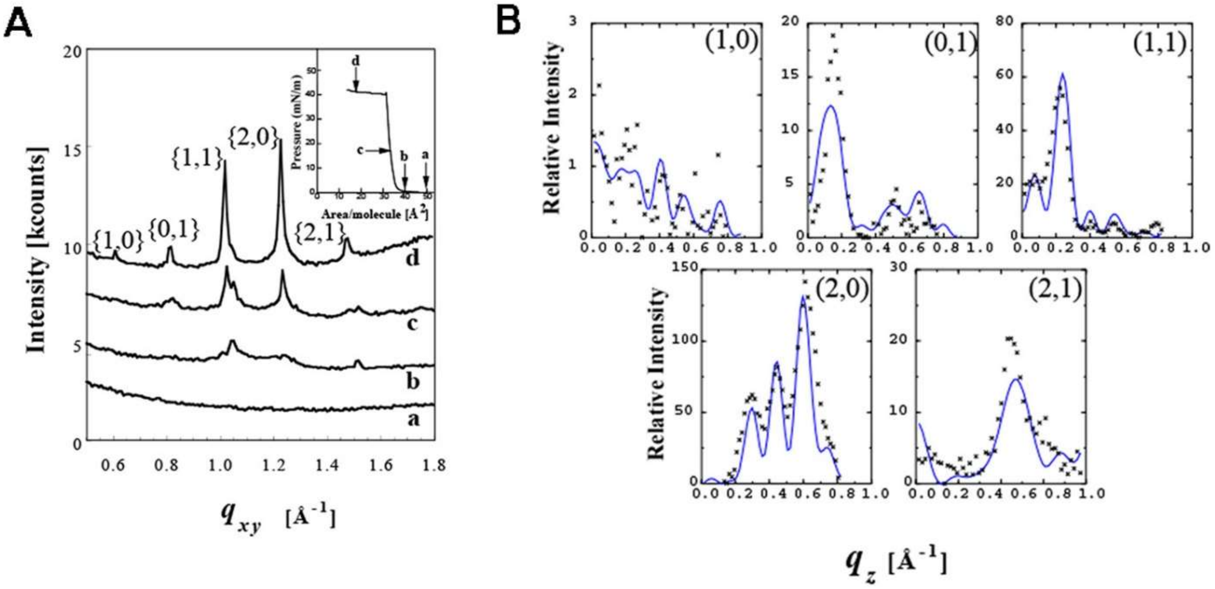

Fig. S14. GIXD patterns of a stigmasterol film at the air-water interface, obtained at 5°C, reproduced from the PhD Thesis of Hanna Rapaport[16] and reference (5). **(A)** The GIXD peaks at increasing surface pressure (a-d) are shown in the inset. The GIXD peak patterns (c) and (d) at 33 Å^2 and 16Å^2, respectively, clearly indicate the formation of crystalline material. The *h,k* indices of the different GIXD peaks, correspond to their Bragg rods shown in panel **(B).** The measured Bragg rods correspond to the GIXD peaks (d). The computed Bragg rod profiles are based on a trilayer model of stigmasterol.

§15. Transformation of the monoclinic form on increased interlayer growth.

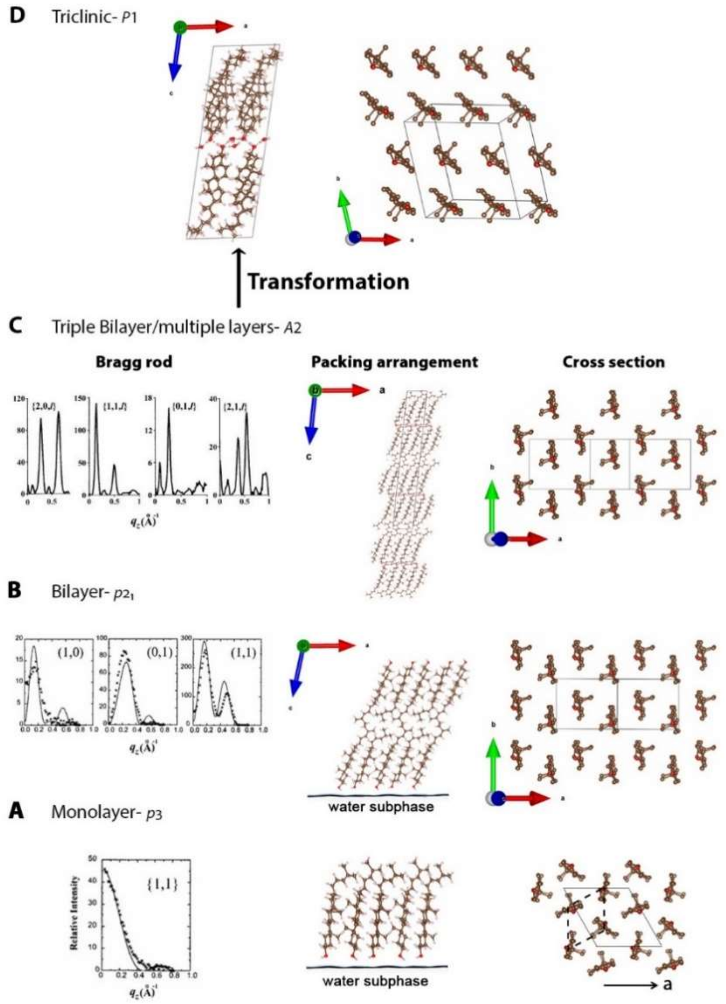

Fig. S15. The different stages of cholesterol formation, at the air-water interface,[3, 5] or hydrated at both sides.[17, 18] **(A)** Crystalline monolayer of cholesterol at the water surface. (Left to right) Bragg rod corresponding to broad single Bragg peak. The profile of this Bragg rod indicates that the long axis of the molecule is aligned perpendicular to the water surface. The full width at half maximum (FWHM) of the Bragg rod indicates that it is a monolayer. The side and top views of the monolayer structure, with proposed *p*3 trigonal symmetry. The molecule undergoes pronounced librational motion about its long axis. The molecules are arranged in an *a'b'* super cell (——) constructed from the subcell (· · ·). **(B)** Crystalline bilayer of cholesterol at the water surface or hydrated at opposite sides. (Left to right) Several {*h,k*} Bragg rod profiles corresponding to different Bragg peaks. The FWHM of the Bragg rods yielded the thickness of the bilayer; Side and top views of the packing arrangement of the crystalline bilayer with lattice symmetry $p2_1$. **(C)** Crystalline triple bilayer of cholesterol monohydrate with lattice symmetry *A*2. (Left to right) Several {*h,k,l*} Bragg rod profiles corresponding to different Bragg peaks. The FWHM of the Bragg rods

yielded the thickness of the film; Packing arrangement of the triple bilayer of cholesterol monohydrate; Cross-section through a cholesterol layer. **(D)** Transformation of crystalline cholesterol.H_2O from monoclinic *A*2[3] to triclinic *P*1[7] symmetry: Part of the packing arrangement viewed edge-on to the bilayer; Cross-section through a layer of cholesterol molecules.

References

1. I Kuzmenko, et al., Design and characterization of crystalline thin film architectures at the air-liquid interface: Simplicity to complexity. *Chem Rev* **101**, 1659–1696 (2001).
2. D Jacquemain, et al., Two-dimensional crystallography of amphiphilic molecules at the air-water interface. *Angew Chem Int Ed* **31**, 130–152 (1992).
3. I Solomonov, MJ Weygand, K Kjaer, H Rapaport, L Leiserowitz, Trapping crystal nucleation of cholesterol monohydrate: relevance to pathological crystallization. *Biophys J* **88**, 1809–1817 (2005).
4. BM Craven, GT DeTitta, Cholesteryl myristate: structures of the crystalline solid and mesophases. *J Chem Soc, Perkin Trans 2*, 814–822 (1976).
5. H Rapaport, et al., Cholesterol monohydrate nucleation in ultrathin films on water. *Biophys J* **81**, 2729–2736 (2001).
6. BM Craven, Cholesterol Crystal Structures: Adducts and Esters. In *Handbook of Lipid Research*, eds. DJ Hanahan, DM Small. (Plenum Press) Vol. 4, pp. 149–182 (1986).
7. BM Craven, Crystal structure of cholesterol monohydrate. *Nature* **260**, 727–729 (1976).
8. BM Craven, Pseudosymmetry in cholesterol monohydrate. *Acta Cryst B* **35**, 1123–1128 (1979).
9. MC Frincu, SD Fleming, AL Rohl, JA Swift, The epitaxial growth of cholesterol crystals from bile solutions on calcite substrates. *J Am Chem Soc* **126**, 7915–7924 (2004).
10. D Weihs, et al., Biliary cholesterol crystallization characterized by single-crystal cryogenic electron diffraction. *J Lipid Res* **46**, 942–948 (2005).
11. Biovia Materials Studio, https://www.3ds.com/products-services/biovia/products/molecular-modeling-simulation/biovia-materials-studio (accessed Feb 15, 2022).
12. SL Mayo, BD Olafson, WA Goddard, Dreiding: a generic force field for molecular simulations. *J Phys Chem* **94**, 8897–8909 (1990).
13. N Varsano, I Fargion, SG Wolf, L Leiserowitz, L Addadi, Formation of 3D cholesterol crystals from 2D nucleation sites in lipid bilayer membranes: implications for atherosclerosis. *J Am Chem Soc* **137**, 1601–1607 (2015).
14. N Varsano, et al., Two polymorphic cholesterol monohydrate crystal structures form in macrophage culture models of atherosclerosis. *PNAS* **115**, 7662–7669 (2018).

15. R-W Jiang, S-C Ma, PP-H But, TCW Mak, New antiviral cassane furanoditerpenes from *Caesalpinia minax*. *J Nat Prod* **64**, 1266–1272 (2001).

16. H Rapaport, PhD thesis, Structural characterization of membrane-active compounds on model films at interfaces (The Weizmann Institute of Science) (1998).

17. R Ziblat, K Kjaer, L Leiserowitz, L Addadi, Structure of cholesterol/lipid ordered domains in monolayers and single hydrated bilayers. *Angew Chem Int Ed* **48**, 8958–8961 (2009).

18. R Ziblat, L Leiserowitz, L Addadi, Crystalline domain structure and cholesterol crystal nucleation in single hydrated DPPC:cholesterol:POPC bilayers. *J Am Chem Soc* **132**, 9920–9927 (2010).
